# Supplementary material for: Measuring context dependency in birdsong using artificial neural networks
Source: PLoS Comput Biol. 2021 Dec 28;17(12):e1009707. doi: 10.1371/journal.pcbi.1009707 (PMC8746767; doi:10.1371/journal.pcbi.1009707)
Supplement: S1 Text — (PDF) [file pcbi.1009707.s001.pdf]

Supporting Information

S1 Details on syllable clustering by VAE

This section is a detailed description of our syllable clustering based on ABCD-VAE (also see [1] for another application of this proposed method). S1.A describes the seq2seq backbone of the VAE, and S1.B explains the ABCD-VAE together with the optimization objective. S1.C defines the parameter settings and the training procedure. S1.D discusses problems with the standard Gaussian VAE and the motivation behind our discrete VAE. S1.E reports the full V-measure evaluation of the alignment between the unsupervised classifications and manual annotations of Bengalese and zebra finch syllables. We also provide the detailed mathematical definition of the metric. Finally, S1.F discusses the robustness of clustering by ABCD-VAE regarding variations in the amplitude and duration of birdsong syllables.

S1.A Seq2Seq autoencoder

Fig A shows the global architecture of our seq2seq VAE, which consists of three modules: the encoder, ABCD-VAE, and the decoder. The entire network receives a time series of syllable spectra, $\mathbf{y} := (\mathbf{y}_1, \dots, \mathbf{y}_T)$, as its input (in the encoder module) and reconstructs the input data. The reconstruction includes the prediction of each spectrum, $\hat{\mathbf{y}} := (\hat{\mathbf{y}}_1, \dots, \hat{\mathbf{y}}_T)$, as well as that of the offset T of the time series, implemented by binary judgments of whether each time step t is the offset ($h_t = 1$) or not ($h_t = 0$). During the reconstruction process, a fixed-dimensional representation of the entire syllable was obtained between the encoder and decoder and was classified into a discrete category by the ABCD-VAE module.

The backbone of the encoder module is the bidirectional LSTM [2, 3]. This RNN processes the input spectra forward and backward. The last hidden and cell states in the two directions are concatenated and transformed by a multi-layer perceptron (MLP).

The MLP output is fed to the ABCD-VAE module (see S1.B) and classified into a discrete category that has a corresponding real-value vector representation. The ABCD-VAE outputs the vector representation of the assigned category, which is concatenated with the embedding of the speaker s of the input syllable. Accordingly, the discrete syllable categories in the ABCD-VAE need not encode speaker characteristics, resulting in speaker normalization [4, 5, 6, 7]. See S1.D for the motivation behind using this speaker normalization for the birdsong data. The concatenation of the output from the ABCD-VAE and the speaker embedding is transformed by another MLP and fed to the decoder LSTM, which is unidirectional. For each time, step $t \in \{1, \dots, T\}$, the output from the LSTM is sent to two distinct MLPs. One of them computes the logits for the offset predictions ($\mathbb{P}(h_t)$). The other MLP parameterizes the isotropic Gaussian probability density function of the spectrum reconstruction [8]. We sampled $\hat{\mathbf{y}}_t$ using this Gaussian, which is used as the input to the LSTM at the next time step $t+1$ (the initial input is $\hat{\mathbf{y}}_0 = 0$).[1]

S1.B ABCD-VAE

This section provides details on the ABCD-VAE. We start with a mathematical description of the model and then move to an explanation of the network implementation. Just like other VAEs, we assumed a prior distribution of the latent feature $z^{(i)}$ of each time-series data i. $z^{(i)}$ is discrete in this study and its prior is the Dirichlet-Categorical distribution. Eq. A and B below define this prior as the two-step generative procedure. The time-series data—represented by the spectra $\mathbf{y}^{(i)}$ and offset judgments $\mathbf{h}^{(i)}$ —are generated conditioned on $z^{(i)}$, whose probability function is implemented by the decoder (Eq. C).

[1] Note that the mathematically correct input to the decoder LSTM is the ground truth spectra, $\mathbf{y}_t$, rather than the reconstruction, $\hat{\mathbf{y}}_t$, because the objective function is the joint probability of $\mathbf{y}$ and $\mathbf{h}$ (see Eq. D). However, the ground truth input to the decoder caused a *uninformative latent variable problem.* The decoder LSTM is considerably powerful and easily trained to fit to the overall distribution of the time-series data while ignoring information from the encoder [9, 10, 11]. We found that our seq2seq VAE did not suffer from this issue when the noisy, reconstructed values $\hat{\mathbf{y}}_t$ were used instead of the ground truth [5].

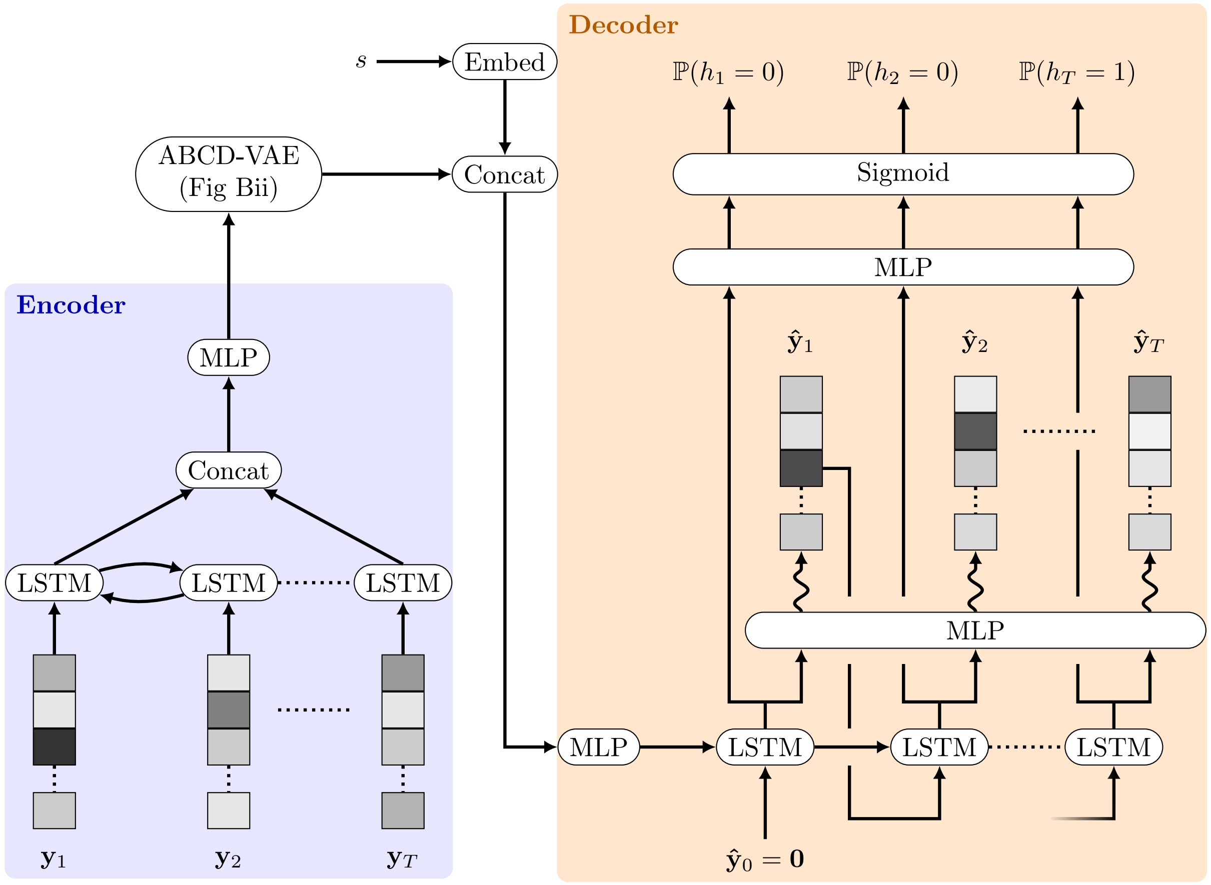

Fig A: The architecture of the RNN-VAE, consisting of the Encoder module and the RNN Decoder module. The wavy arrows represent isotropic Gaussian sampling parameterized by the output of the previous MLP.

$$\boldsymbol{\pi} \sim \text{Dirichlet}(\boldsymbol{\alpha}) \tag{A}$$

$$z^{(i)} \mid \boldsymbol{\pi} \sim \text{Categorical}(\boldsymbol{\pi}) \tag{B}$$

$$(\mathbf{y}^{(i)}, \mathbf{h}^{(i)}) \mid z^{(i)} \sim p(\cdot \mid z^{(i)}, s^{(i)}) = \text{Decoder}(z^{(i)}, s^{(i)}) \tag{C}$$

Where $\boldsymbol{\alpha} := (\alpha_1, \ldots, \alpha_K)$ are positive real numbers and $\boldsymbol{\pi} := (\pi_1, \ldots, \pi_K) \in \Delta^{K-1}$ is a probability vector (i.e., $\forall k \in \{1, \ldots, K\}, \pi_k \geq 1 \wedge \sum_{k=1}^{K} \pi_k = 1$). The Dirichlet-Categorical prior causes a rich-gets-richer bias, preferring a smaller number of categories to be used repeatedly [12, 13, 14], while the (uniform) categorical prior—standard in the categorical VAE [15]—eats up all the categories available. Because of this Occam's razor effect, the Dirichlet-Categorical prior (and its extension to unbounded choices, the Dirichlet process) is popular in Bayesian learning when the model needs to detect the appropriate number of categories in the posterior (see [16, 17, 18, 19, 20, 21, 22, 23, 24] for examples in computational linguistics and cognitive science). The role of the encoder is to approximate the posterior $p(\boldsymbol{\pi}, \mathbf{z} \mid \mathbf{y}, \mathbf{h})$ of the model in Eq. A-C. We make several assumptions on the approximated posterior, denoted by $q(\boldsymbol{\pi}, \mathbf{z} \mid \mathbf{y})$.[2]

1. $\boldsymbol{\pi}$ and $\mathbf{z}$ are independent given $\mathbf{y}$ in q: i.e., $q(\boldsymbol{\pi}, \mathbf{z} \mid \mathbf{y}) = q(\boldsymbol{\pi} \mid \mathbf{y}) q(\mathbf{z} \mid \mathbf{y})$.
2. Each $z^{(i)}$ is the categorical distribution and is independent of the other $z^{(j)}$ ($i \neq j$) given the corresponding data $\mathbf{y}^{(i)}$.

[2] Note that the time series input into the encoder contains implicit information about its length/offset, $\mathbf{h}^{(h)}$.

3. $\boldsymbol{\pi}$ is independent of $\mathbf{y}$ in q: i.e., $q(\boldsymbol{\pi} \mid \mathbf{y}) = q(\boldsymbol{\pi})$.

4. $q(\boldsymbol{\pi})$ is the Dirichlet distribution whose parameters are in the form of $\boldsymbol{\omega} := N\boldsymbol{\theta} + \alpha$, where N is the data size (the total number of time-series data) and $\boldsymbol{\theta}$ is a trainable vector in the simplex (i.e., $\forall k \in \{1, \dots, K\}, \theta_k \geq 0 \wedge \sum_k \theta_k = 1$).

The assumptions in 1 and 3 are imported from the mean-field variational inference, and 3 provides the optimal form of $q(\boldsymbol{\pi})$ under the assumptions [12].[3] The optimization objective of the entire VAE is the maximization of the evidential lower bound (ELBO) of the log marginal likelihood $\log p(\mathbf{y}, \mathbf{h})$ [8, 9].

$$\begin{aligned} \log p(\mathbf{y}, \mathbf{h}) &\geq \log p(\mathbf{y}, \mathbf{h}) - D_{\mathrm{KL}} \left[q(\boldsymbol{\pi}, \mathbf{z} \mid \mathbf{y}) \parallel p(\boldsymbol{\pi}, \mathbf{z} \mid \mathbf{y}, \mathbf{h}) \right] \\ &= - D_{\mathrm{KL}} \left[q(\boldsymbol{\pi}, \mathbf{z} \mid \mathbf{y}) \parallel p(\boldsymbol{\pi}, \mathbf{z}) \right] + \mathrm{E}_q \left[\log p(\mathbf{y}, \mathbf{h} \mid \mathbf{z}) \right] \\ &=: \mathrm{ELBO} \end{aligned} \tag{D}$$

Based on the assumptions of the approximated posterior q, the first term in Eq. D is rewritten as follows:

$$D_{\mathrm{KL}} \left[q(\boldsymbol{\pi}, \mathbf{z} \mid \mathbf{y}) \parallel p(\boldsymbol{\pi}, \mathbf{z}) \right] = \mathrm{E}_q \left[\log q(\boldsymbol{\pi}) \right] - \mathrm{E}_q \left[\log p(\boldsymbol{\pi}) \right] + \sum_{i=1}^{N} \left(\mathrm{E}_q \left[\log q(z^{(i)} \mid \mathbf{y}^{(i)}) \right] - \mathrm{E}_q \left[\log p(z^{(i)} \mid \boldsymbol{\pi}) \right] \right)$$

Where each term has a closed form. During the mini-batch learning, the first two terms outside the summation operation, the index i of which denotes data, are multiplied by B/N, where B is the batch size. For the second term in Eq. D, $\mathrm{E}_q \left[\log p(\mathbf{y}, \mathbf{h} \mid \mathbf{z}) \right] = \sum_{i=1}^{N} \mathrm{E}_q \left[\log p(\mathbf{y}^{(i)}, \mathbf{h}^{(i)} \mid z^{(i)}) \right]$, we approximate the computation of the expectation using the Monte Carlo method [8]. We adopted the Gumbel-Softmax approximation proposed in [15] because the exact sampling from the categorical distribution $q(z^{(i)} \mid \mathbf{y}^{i})$ is incompatible with gradient-based training.

$$\mathrm{E}_q \left[\log p(\mathbf{y}^{(i)}, \mathbf{h}^{(i)} \mid z^{(i)}) \right] \approx \log p(\mathbf{y}^{(i)}, \mathbf{h}^{(i)} \mid \tilde{\mathbf{z}}^{(i)}) \qquad (\tilde{\mathbf{z}}^{(i)} \in \Delta^{K-1} : \text{Sample from Gumbel-Softmax})$$

To simplify the learning process, the linear transformation before and after the Gumbel-Softmax sampling share the same weight matrix $\mathbf{M}$. The linear transformation before the Gumbel-Softmax computes the logits by multiplying the output of the encoder with $\mathbf{M}$, while the transformation after the Gumbel-Softmax computes $\tilde{\mathbf{z}}^{(i)} \mathbf{M}^{\mathrm{T}}$ (Fig Bi).[4] In other words, $\mathbf{M}$ is the "codebook" whose column vectors are the real-value representation of the corresponding discrete categories. The first linear transformation computes the similarity between the encoder output and each column vector, and the second transformation picks up the column vector of the sampled category (assuming that the Gumbel-Softmax sample $\tilde{\mathbf{z}}^{(i)}$ is close to a one-hot vector). Thus, our VAE is similar to the vector-quantized VAE [4, 5, 7], which uses the L2-similarity instead of our unnormalized cosine similarity (without the randomness).

The exact implementation of the ABCD-VAE is based on the scaled dot-product attention used in the Transformer [25, 26] and depicted in Fig Bii. The attention mechanism first computes the dot product of the encoder's output (query) and the codebook $\mathbf{M}$ (memory; i.e., both key and value), yielding the similarity between the two. This similarity is scaled by $\sqrt{D_h}$, where D_h is the dimensionality of the encoder output and the column vectors of the codebook (i.e., the number of rows in $\mathbf{M}$). The scaled similarity is transformed by the softmax into the posterior probability vector $q(z^{(i)} \mid \mathbf{y}^{(i)})$, and a Gumbel-Softmax sample $\tilde{\mathbf{z}}^{(i)}$ is drawn from this probability distribution. Finally, $\tilde{\mathbf{z}}^{(i)}$ is multiplied by $\mathbf{M}^{\mathrm{T}}$ (used as the value) and sent to the decoder.

[3] The optimal update of $\boldsymbol{\theta}$ is proportional to the expected number of data classified into each category [12]. However, the exact computation of these expected counts requires iterations over all data and is inefficient. Therefore, we train $\boldsymbol{\theta}$ using the gradient ascent.

[4] For simplicity, the linear transformations do not have bias terms.

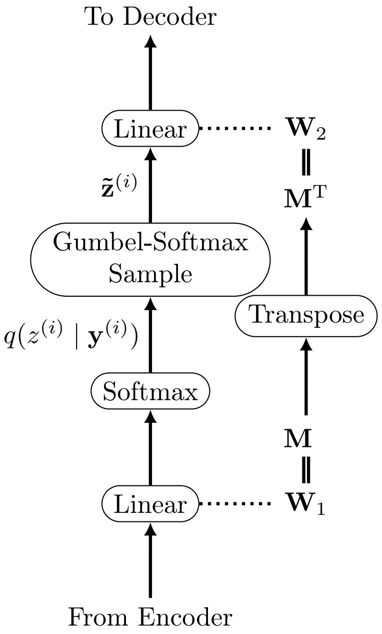

(i) Shared Weight Matrix

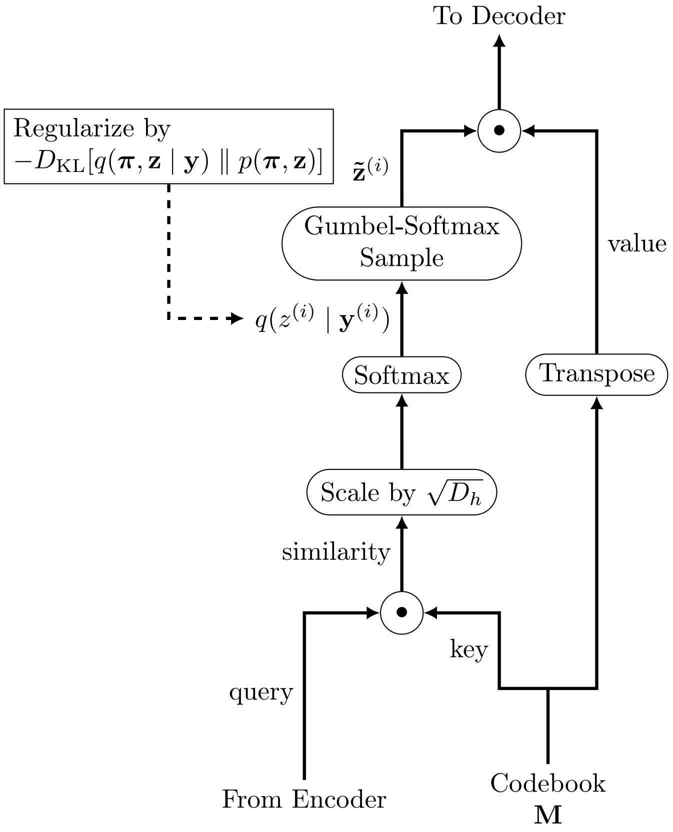

(ii) Attention-based Implementation

Fig B: ABCD-VAE.

S1.C Parameter settings and training procedure

The input time series $\mathbf{y}_1, \ldots, \mathbf{T}$ were obtained as follows. We first applied the short-time Fourier transform to the recordings having the 8 msec Hanning window and 4 msec stride (i.e., 256 samples for the window length and 128 samples for the step size because the sampling rate was 32 kHz). The spectral amplitude was then log-transformed (after 2^{-15} was added to avoid underflow) and rescaled by 11^{-1} ($> \log 2^{15}$).

All the hidden states in the VAE had the dimensionality $D_h = 256$. The number of possible discrete categories (i.e., upper bound) was $K = 128$ and the non-linearity of the MLPs was tanh. The neural network was trained by the stochastic gradient ascent for 20 epochs. The first five epochs were used for "pretraining" where the posterior probability $q(z^{(i)} \mid \mathbf{y}^{(i)})$ was multiplied with the codebook $\mathbf{M}$ without sampling from the Gumbel-Softmax distribution. After this process, the temperature τ of the Gumbel-Softmax was annealed every 1000 iterations according to the schedule $\tau = \exp(-10^{-5}m)$, where m is the number of iterations after the initial five epochs [15]. The learning rate was initially set as 1.0 and multiplied by 0.1 after every epoch in which validation loss was not improved. The gradient norms were clipped at 1.0 to avoid an explosion. No dropout or momentum were introduced.

S1.D Problems with the Gaussian VAE analysis of birdsong

This section discusses the real-value features and problems of birdsong syllables obtained by the standard Gaussian VAE. The Gaussian VAE is a popular way of obtaining real-valued features of data in an arbitrary dimensional space [8] and has been used for analyses of animal vocalization [27, 28, 29]. We also tested it on our birdsong data and reported clustering results based on the acoustic features extracted from it as baselines (see Table 1 in the main text). The syllable features of the Gaussian VAE exhibited some clear clusters when we looked at each individual bird separately (Fig Ci, the original features had 16 dimensions, which we embedded into the two-dimensional space by tSNE for visualization [30]).

When we represent syllables of multiple individuals, however, we no longer see such clear clusters. Fig Cii shows the syllable features of all the 18 Bengalese finches used in this study (distinguished by the colors and shapes of the markers). Many syllables are confused around the center, and tiny clusters in the peripheral are individual-specific.

We also tried the speaker normalization technique used in discrete VAEs [4, 5, 7], feeding the speaker ID the decoder module (Fig A, wherein the speaker embeddings were jointly trained with the other modules; cf. [31]), but this did not solve the problem (Fig Ciii); GMM clustering in the resulting feature space aligned poorly with human annotations when the number of syllable categories was manually specified as 14 (= the annotation labels) and 37 (= the ABCD-VAE result), and resorted to the fine-grained, speaker-specific classification when the number of categories was auto-detected (≥ 128; Table A). The speaker normalization increased the speaker perplexity only when the number of syllable categories was fixed as moderate numbers. That is, the speaker normalization only removed the global speaker-based zoning of the latent space, and individuality was still visible locally to the extent that the uncertainty of speaker identification from a 128-wise classification was less than a flip of a coin. Given the inappropriate distribution of the syllable features encoded by the Gaussian VAE (but see [31, 32] for other individual-normalization techniques that work on continuous spaces), we adopted end-to-end clustering with the ABCD-VAE.

S1.E V-measure metric for evaluation of clustering results

In the main text, we evaluated the clustering results of birdsong syllables by their alignment with human annotations measured by Cohen's Kappa coefficients and homogeneity. These two metrics examined whether clustered syllables were annotated with the same label. On the other hand, they did not penalize overclassification. In our study, we compared the baseline and topline results with different numbers of syllable categories, so our evaluation did not take into account overclassification. In more general situations, however, overclassification is an important aspect of clustering and thus must be penalized. One option for a more comprehensive metric of clustering quality is *V-measure*, which combines homogeneity with another submetric for scoring overclassification, called *completeness*. Completeness evaluates clustering results in the opposite direction from homogeneity: it requires syllables annotated with the same label to belong to the same model-predicted category. For example, suppose that syllables labeled as "a" are split into two categories, "A" and "B", by clustering. As explained in the main text, homogeneity is satisfied even under

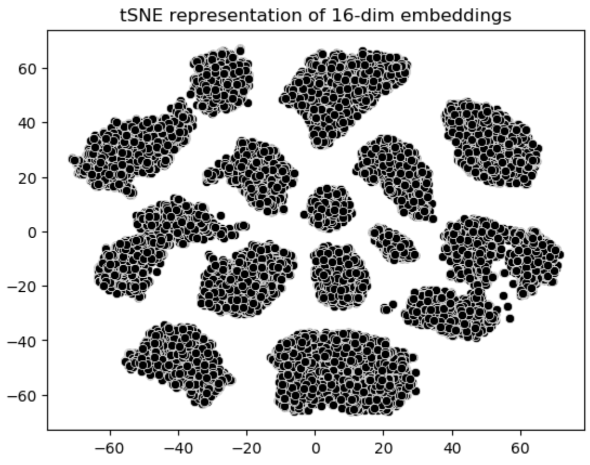

(i) Single Individual (b03)

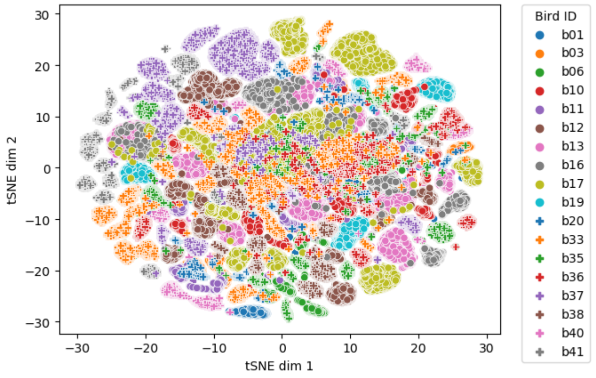

(ii) All Individuals

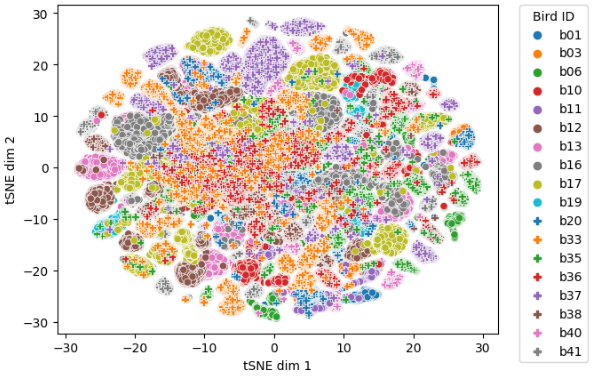

(iii) Speaker-Normalized

Fig C: Continuous-valued encodings of Bengalese finch syllables by Gaussian VAE. The original dimensionality of the features was 16, and they were embedded into the two-dimensional space by tSNE. (i) Syllables from a single individual (b03). (ii) Syllables from all the individuals were plotted together, using colors and shapes of the markers to distinguish the individuals. (iii) Syllables from all the individuals encoded with the speaker normalization technique used in discrete VAEs.

Table A: Quantitative evaluation of the clustering by the ABCD- vs. Gauss-VAE for Bengalese finch syllables, extending Table 1 with the results based on the speaker-normalized Gauss-VAE. Cohen's kappa coefficient and homogeneity evaluated the alignment of the discovered clusters with manual annotations by a human expert. These scores for each individual bird were computed separately and their mean, maximum, and minimum over the individuals were reported since the manual annotation was not shared across individuals (see Materials and methods). Additionally, the perplexity of individual identification scored the amount of individuality included in the syllable categories yielded by the ABCD- and Gauss-VAE. The best scores are in boldface (results under the all-birds-together and bird-specific settings were ranked separately).

Data	Method	# of clusters (source)	Cohen's Kappa mean [min,max]	Homogeneity mean [min,max]	Speaker Perplexity
All-Birds-Together	ABCD-VAE	37	**0.8990** [**0.7740**, **0.9929**]	**0.9084** [**0.7635**, 0.9868]	8.0434
All-Birds-Together	Gauss-VAE + GMM	37 (ABCD-VAE)	0.7446 [0.5956, 0.8912]	0.7844 [0.6004, 0.9086]	4.0783
All-Birds-Together	Gauss-VAE + GMM	14 (manual)	0.6057 [0.4250, 0.8972]	0.6718 [0.5053, 0.8536]	6.7212
All-Birds-Together	Gauss-VAE + GMM	≥128 (auto-detected)	0.8475 [0.5725, 0.9911]	0.8773 [0.6666, 0.9869]	1.7112
All-Birds-Together	Gauss-VAE + GMM + Speaker-Normalization	37 (ABCD-VAE)	0.6403 [0.4410, 0.8471]	0.6529 [0.4714, 0.8367]	9.0148
All-Birds-Together	Gauss-VAE + GMM + Speaker-Normalization	14 (manual)	0.4820 [0.2787, 0.6497]	0.4975 [0.2863, 0.6600]	**11.3122**
All-Birds-Together	Gauss-VAE + GMM + Speaker-Normalization	≥128 (auto-detected)	0.8649 [0.5320, 0.9885]	0.8754 [0.5800, **0.9904**]	1.9887
Bird-Specific	Gauss-VAE + GMM	37 (ABCD-VAE)	0.9304 [0.6619, 0.9906]	0.9292 [0.6479, 0.9893]	—
Bird-Specific	Gauss-VAE + GMM	5–14 (manual)	0.7888 [0.5012, 0.9328]	0.8090 [0.4732, 0.9254]	—
Bird-Specific	Gauss-VAE + GMM	50–109 (auto-detected)	0.9516 [0.7629, 0.9982]	0.9505 [0.7687, 0.9962]	—
Bird-Specific	Gauss-VAE + GMM + Speaker-Normalization	37 (ABCD-VAE)	0.9137 [0.6052, 0.9893]	0.9152 [0.5952, 0.9893]	—
Bird-Specific	Gauss-VAE + GMM + Speaker-Normalization	5–14 (manual)	0.7641 [0.3636, 0.9271]	0.7889 [0.4271, 0.9401]	—
Bird-Specific	Gauss-VAE + GMM + Speaker-Normalization	61–104 (auto-detected)	**0.9560** [**0.8046**, **0.9943**]	**0.9557** [**0.8011**, 0.9949]	—

this overclassification, as long as all the syllables in "A" and "B" are labeled as "a" and nothing else. By contrast, the classification violates completeness and, thus, it complements homogeneity as an evaluation metric. (Note that completeness does not penalize non-uniformity of clustered syllables regarding human annotations, which violates homogeneity; e.g., it is satisfied even when the model-predicted category "A" includes syllables annotated with different labels, say "a" and "b", as long as all the "a" and "b" syllables belong to "A" and nowhere else.) Mathematically, violation of completeness is defined by the conditional entropy of the distribution of predicted clusters $\mathcal{K}$ given the ground truth classes $\mathcal{C}$:

$$\text{completeness}(\mathcal{C}, \mathcal{K}) := \begin{cases} 1 & H(\mathcal{K}) = 1 \\ 1 - \frac{H(\mathcal{K}|\mathcal{C})}{H(\mathcal{K})} & \text{Otherwise} \end{cases}$$

Note that the non-conditional entropy $H(\mathcal{K})$ is the normalizing term that makes completeness range between 0 and 1. Finally, V-measure is defined by the harmonic mean of homogeneity and completeness:

$$\text{V-measure}(\mathcal{C}, \mathcal{K}) := \frac{2 * \text{homogeneity}(\mathcal{C}, \mathcal{K}) * \text{completeness}(\mathcal{C}, \mathcal{K})}{\text{homogeneity}(\mathcal{C}, \mathcal{K}) + \text{completeness}(\mathcal{C}, \mathcal{K})}$$

Table B reports the completeness and V-measure scores of the syllable clustering results. We also repeat homogeneity in Table 1 for easier comparison among scores.

S1.F Classification confidence against variations in duration and amplitude

This section discusses the robustness of clustering by the ABCD-VAE regarding variations in the amplitude and duration of birdsong syllables. Figs D–G plots the probability of MAP syllable categories of Bengalese finch (D, E) and zebra finch (F, G) against the maximum amplitude (D, F) and duration (E, G) of the syllables. We can see no general pattern such as "classification probability decreases (i.e., the model gets less confident) as the amplitude/duration diverges from the mean". Instead, different categories use amplitude/duration information differently, some exhibiting a tendency to assign a greater probability to shorter/longer or louder/softer syllables while the others are more robust against variations in amplitude/duration. Overall, the Pearson correlation coefficient between the MAP classification probability and the deviation of the amplitude/duration from the median (all in log scale) was small: -0.0791 (vs. amplitude) and 0.0559 (vs. duration) for Bengalese finch; 0.1217 (vs. amplitude) and 0.0328 (vs. duration) for zebra finch. It also is certain that none of the detected syllable categories was purely characterized by amplitude nor duration; syllables having the same amplitude/duration were assigned various classification probabilities, indicating the existence of other acoustic factors that have an effect on the classification.

We conclude this section by introducing a possible method to ignore variations in amplitude/duration as noise. Remember that the learning objective of the canonical VAE is to reconstruct each input syllable as precisely as possible, and we adopted this framework in the present study. However, one can also add some noise to the input—including manipulation of amplitude or duration [33]—and train a *denoising* VAE to recover the original data [34]. Then, the VAE will ignore variations in the manipulated acoustic dimension as noise rather than the systematic differences among categories.

Table B: Scores of the clustering by the ABCD-VAE. Homogeneity, completeness, and V-measure evaluated the alignment of the discovered clusters with manual annotations by a human expert. These scores for each individual bird were computed separately and their mean, maximum, and minimum over the individuals were reported since the manual annotation was not shared across individuals (see Materials and methods). The best scores are in boldface (results under the all-birds-together and bird-specific settings were ranked separately).

Species	Method	# of clusters (source)	Homogeneity mean [min,max]	Completeness mean [min,max]	V-measure mean [min,max]
BF	ABCD-VAE	37	**0.9084** [**0.7635**, 0.9868]	0.6859 [0.5069, 0.8468]	0.7765 [**0.6575**, 0.8797]
	Gauss-VAE + GMM (All-Birds-Together)	37 (ABCD-VAE)	0.7844 [0.6004, 0.9086]	0.7595 [0.5555, 0.9190]	0.7679 [0.6105, **0.8940**]
		14 (manual)	0.6718 [0.5053, 0.8536]	**0.8249** [**0.6503**, **0.9492**]	0.7353 [0.6158, 0.8489]
		≥128 (auto-detected)	0.8773 [0.6666, **0.9869**]	0.7177 [0.5499, 0.8652]	**0.7837** [0.6432, 0.8743]
	Gauss-VAE + GMM (Bird-Specific)	37 (ABCD-VAE)	0.9292 [0.6479, 0.9893]	0.5890 [0.4058, 0.7522]	0.7148 [0.5750, 0.8494]
		5–14 (manual)	0.8090 [0.4732, 0.9254]	**0.8038** [**0.5771**, **0.9216**]	**0.8042** [0.5255, **0.9235**]
		50–109 (auto-detected)	**0.9505** [**0.7687**, **0.9962**]	0.6076 [0.4284, 0.7712]	0.7357 [**0.5965**, 0.8647]
ZF	ABCD-VAE	17	0.6793 [0.4972, 0.8718]	0.6141 [0.3346, 0.8495]	0.6378 [0.4189, 0.8605]
	Gauss-VAE + GMM (All-Birds-Together)	17 (ABCD-VAE)	0.6177 [0.3030, 0.8942]	0.7351 [0.4159, 0.9461]	0.6502 [0.3638, 0.8279]
		13 (manual)	0.6315 [0.0433, 0.9609]	**0.7569** [0.4104, 0.9494]	0.6687 [0.0793, 0.9330]
		≥128 (auto-detected)	**0.9016** [**0.7643**, **0.9894**]	0.7542 [**0.4514**, **0.9935**]	**0.8118** [**0.6173**, **0.9511**]
	Gauss-VAE + GMM (Bird-Specific)	17 (ABCD-VAE)	0.9545 [0.8828, 0.9905]	0.5792 [0.2983, 0.7835]	0.7132 [0.4566, 0.8453]
		4–13 (manual)	0.8623 [0.7056, 0.9607]	**0.7911** [**0.4526**, **0.9886**]	**0.8219** [**0.5858**, **0.9226**]
		18–47 (auto-detected)	**0.9782** [**0.9274**, **1.0000**]	0.5587 [0.3172, 0.7408]	0.7039 [0.4781, 0.8359]

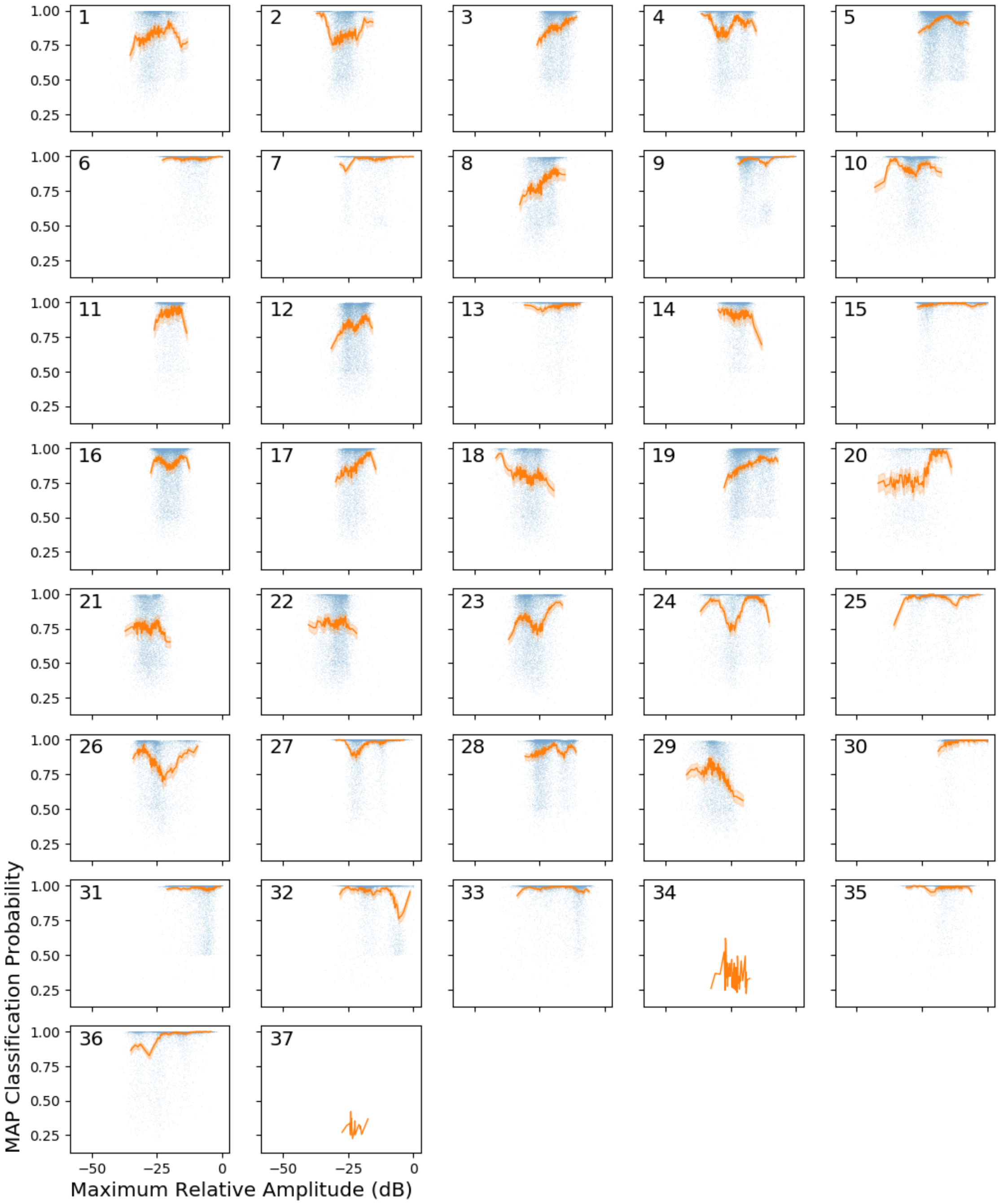

Fig D: Probability of MAP syllable categories (indexed by the integer in the upper right) in Bengalese finch song against maximum amplitude of the syllables. The blue scatter represents individual syllables. The orange lines represent the mean probability over 1%tile windows.

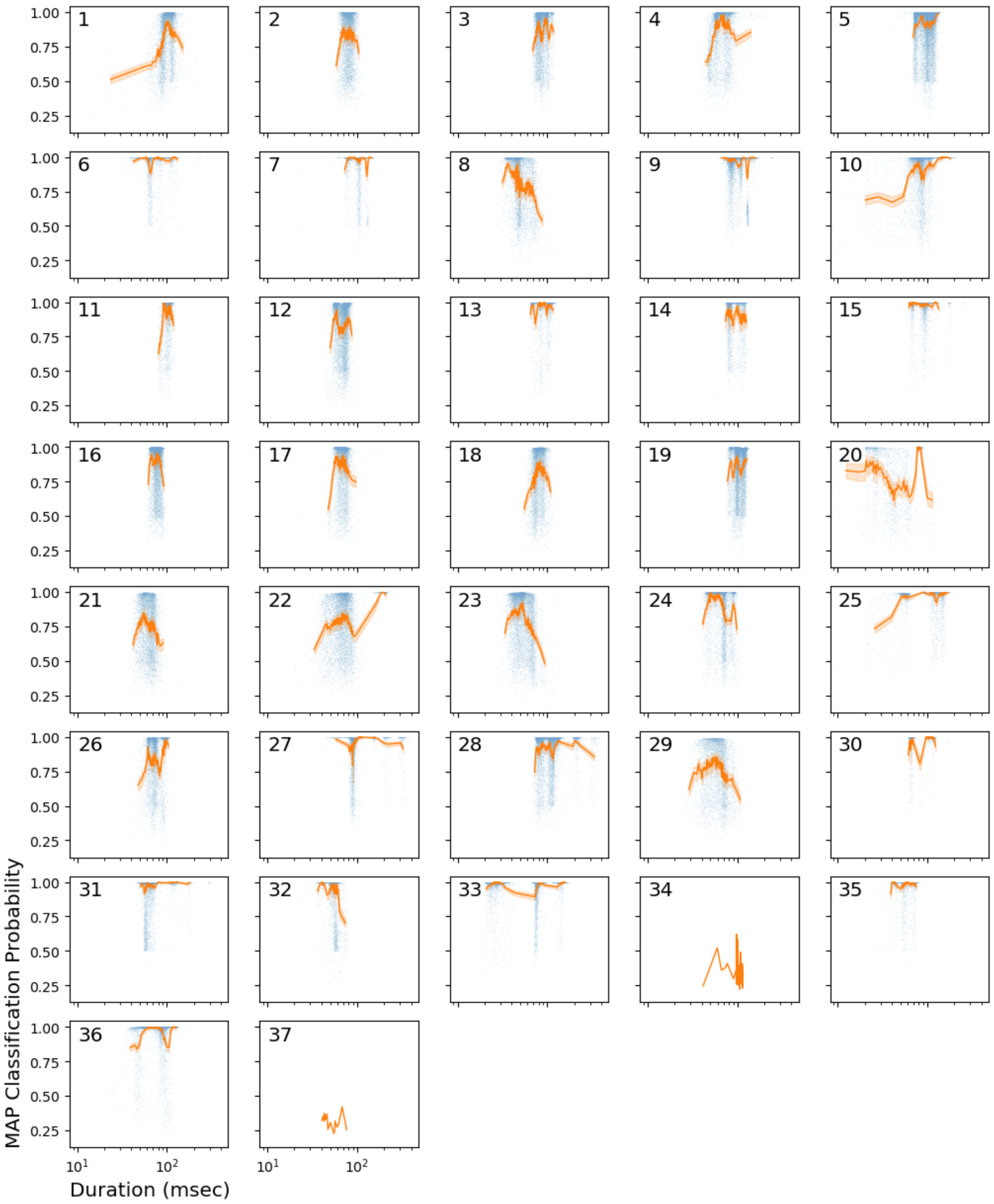

Fig E: Probability of MAP syllable categories (indexed by the integer in the upper right) in Bengalese finch song against syllable duration. The blue scatter represents individual syllables. The orange lines represent the mean probability over 1%tile windows.

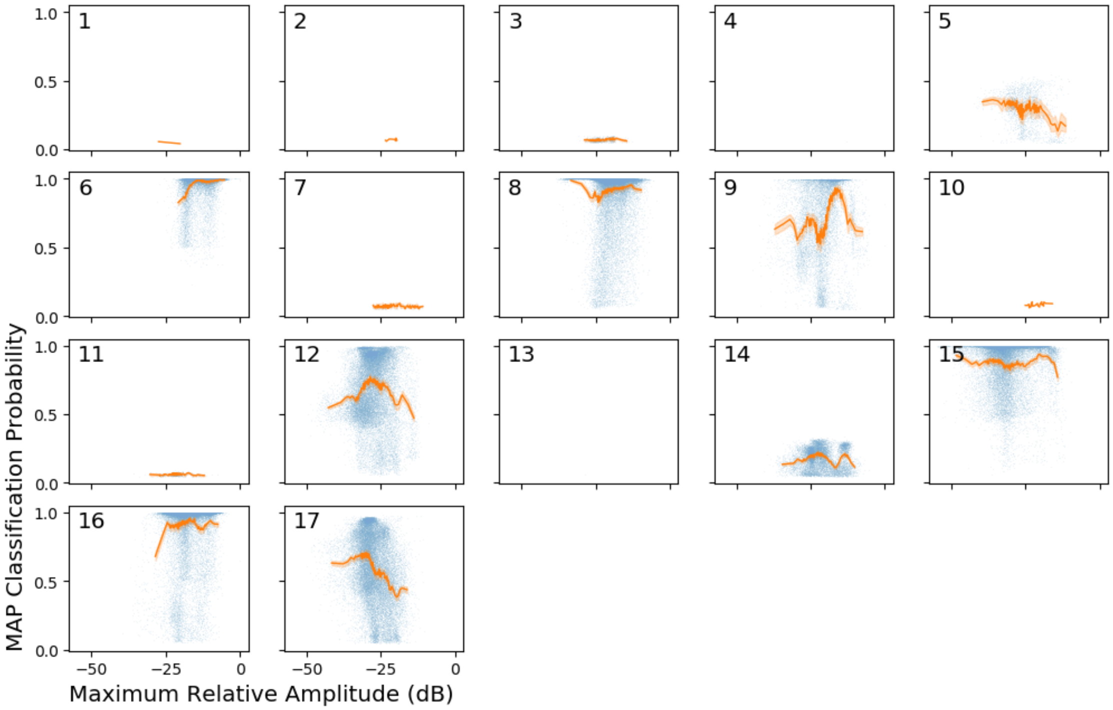

Fig F: Probability of MAP syllable categories (indexed by the integer in the upper right) in zebra finch song against maximum amplitude of the syllables. The blue scatter represents individual syllables. The orange lines represent the mean probability over 1%tile windows. Note that categories numbered 4 and 13 were singletons and thus left empty.

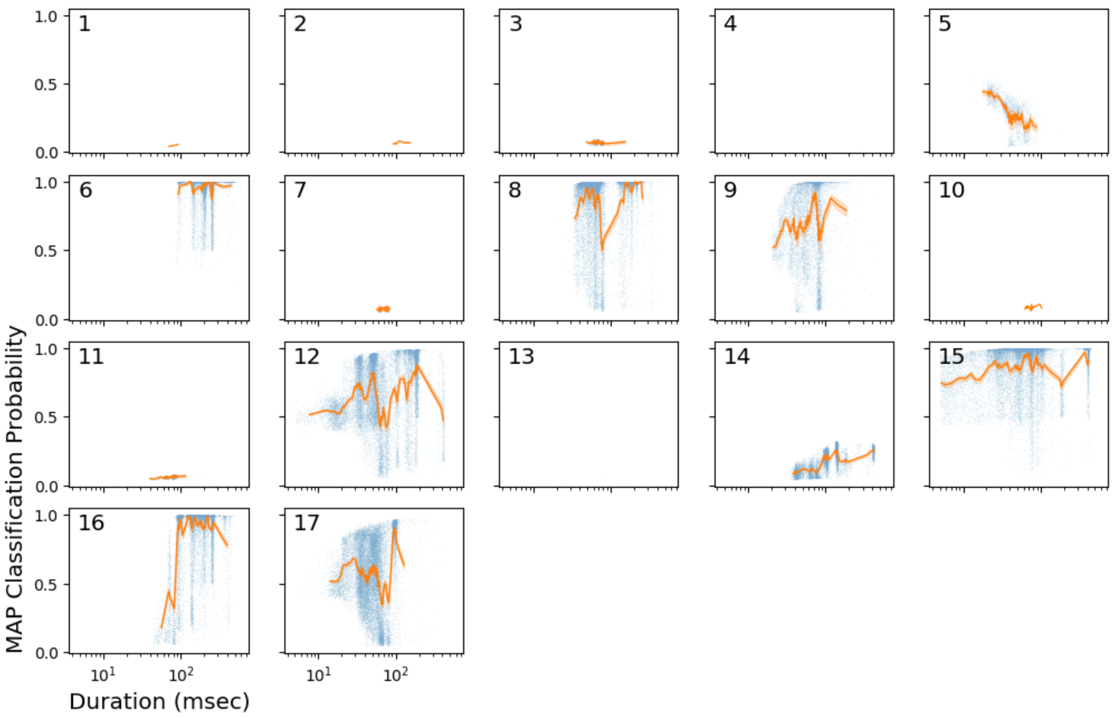

Fig G: Probability of MAP syllable categories (indexed by the integer in the upper right) in zebra finch song against syllable duration. The blue scatter represents individual syllables. The orange lines represent the mean probability over 1%tile windows. Note that categories numbered 4 and 13 were singletons and thus left empty.

References

1. Morita T, Koda H. Exploring TTS without T Using Biologically/Psychologically Motivated Neural Network Modules (ZeroSpeech 2020). In: Proceedings of Interspeech 2020; 2020. p. 4856–4860.

2. Hochreiter S, Schmidhuber J. Long short-term memory. Neural Computation. 1997;9(8):1735–1780. doi:10.1162/neco.1997.9.8.1735.

3. Schuster M, Paliwal KK. Bidirectional recurrent neural networks. IEEE Transactions on Signal Processing. 1997;45(11):2673–2681. doi:10.1109/78.650093.

4. van den Oord A, Vinyals O, Kavukcuoglu K. Neural Discrete Representation Learning. In: Guyon I, Luxburg UV, Bengio S, Wallach H, Fergus R, Vishwanathan S, et al., editors. Advances in Neural Information Processing Systems 30. Curran Associates, Inc.; 2017. p. 6306–6315.

5. Chorowski J, Weiss RJ, Bengio S, van den Oord A. Unsupervised Speech Representation Learning Using WaveNet Autoencoders. IEEE/ACM Transactions on Audio, Speech, and Language Processing. 2019;27(12):2041–2053. doi:10.1109/TASLP.2019.2938863.

6. Dunbar E, Algayres R, Karadayi J, Bernard M, Benjumea J, Cao XN, et al. The Zero Resource Speech Challenge 2019: TTS Without T. In: Proceedings of Interspeech 2019; 2019. p. 1088–1092.

7. Tjandra A, Sisman B, Zhang M, Sakti S, Li H, Nakamura S. VQVAE Unsupervised Unit Discovery and Multi-scale Code2Spec Inverter for Zerospeech Challenge 2019. In: Proceedings of Interspeech 2019; 2019. p. 1118–1122.

8. Kingma DP, Welling M. Auto-Encoding Variational Bayes; 2014. The International Conference on Learning Representations (ICLR) 2014.

9. Bowman SR, Vilnis L, Vinyals O, Dai A, Jozefowicz R, Bengio S. Generating Sentences from a Continuous Space. In: Proceedings of The 20th SIGNLL Conference on Computational Natural Language Learning; 2016. p. 10–21.

10. Zhao T, Zhao R, Eskenazi M. Learning Discourse-level Diversity for Neural Dialog Models using Conditional Variational Autoencoders. In: Proceedings of the 55th Annual Meeting of the Association for Computational Linguistics (Volume 1: Long Papers). Association for Computational Linguistics; 2017. p. 654–664.

11. Liu D, Xue Y, He F, Chen Y, Lv J. μ-Forcing: Training Variational Recurrent Autoencoders for Text Generation. ACM Transactions on Asian Low-Resource Language Information Processing. 2019;19(1). doi:10.1145/3341110.

12. Bishop CM. Pattern recognition and machine learning. Information science and statistics. New York: Springer; 2006.

13. O'Donnell TJ. Productivity and reuse in language : a theory of linguistic computation and storage. Cambridge, MA; London, England: MIT Press; 2015.

14. Little MA. Machine Learning for Signal Processing: Data Science, Algorithms, and Computational Statistics. Oxford University Press; 2019.

15. Jang E, Gu S, Poole B. Categorical Reparameterization with Gumbel-Softmax. In: Proceedings of the 5th International Conference on Learning Representations (ICLR); 2017.

16. Anderson JR. The adaptive character of thought. Studies in cognition. Hillsdale, NJ: L. Erlbaum Associates; 1990.

17. Kurihara K, Sato T. An Application of the Variational Bayesian Approach to Probabilistic Context-Free Grammars. In: International Joint Conference on Natural Language Processing Workshop Beyond Shallow Analyses; 2004.

18. Kurihara K, Sato T. Variational Bayesian Grammar Induction for Natural Language. In: Sakakibara Y, Kobayashi S, Sato K, Nishino T, Tomita E, editors. Grammatical Inference: Algorithms and Applications: 8th International Colloquium, ICGI 2006, Tokyo, Japan, September 20-22, 2006. Proceedings. Berlin, Heidelberg: Springer Berlin Heidelberg; 2006. p. 84–96.

19. Teh YW, Jordan MI, Beal MJ, Blei DM. Hierarchical Dirichlet Processes. Journal of the American Statistical Association. 2006;101(476):1566–1581.

20. Kemp C, Perfors A, Tenenbaum J. Learning overhypotheses with hierarchical Bayesian models. Developmental Science. 2007;10(3):307–321.

21. Goldwater S, L Griffiths T, Johnson M. A Bayesian Framework for Word Segmentation: Exploring the Effects of Context. Cognition. 2009;112:21–54.

22. Feldman NH, Goldwater S, Griffiths TL, Morgan JL. A Role for the Developing Lexicon in Phonetic Category Acquisition. Psychological Review. 2013;120(4):751–778.

23. Kamper H, Jansen A, Goldwater S. A segmental framework for fully-unsupervised large-vocabulary speech recognition. Computer Speech & Language. 2017;46:154–174. doi:10.1016/j.csl.2017.04.008.

24. Morita T, O'Donnell TJ. Statistical Evidence for learnable lexical subclasses in Japanese. Linguistic Inquiry. To appear;doi:10.1162/ling_a_00401.

25. Vaswani A, Shazeer N, Parmar N, Uszkoreit J, Jones L, Gomez AN, et al. Attention is All you Need. In: Guyon I, Luxburg UV, Bengio S, Wallach H, Fergus R, Vishwanathan S, et al., editors. Advances in Neural Information Processing Systems 30. Curran Associates, Inc.; 2017. p. 5998–6008.

26. Devlin J, Chang MW, Lee K, Toutanova K. BERT: Pre-training of Deep Bidirectional Transformers for Language Understanding; 2018. arXiv:1810.04805.

27. Coffey KR, Marx RG, Neumaier JF. DeepSqueak: a deep learning-based system for detection and analysis of ultrasonic vocalizations. Neuropsychopharmacology. 2019;44(5):859–868. doi:10.1038/s41386-018-0303-6.

28. Goffinet J, Mooney R, Pearson J. Inferring low-dimensional latent descriptions of animal vocalizations. bioRxiv. 2019;doi:10.1101/811661.

29. Sainburg T, Thielk M, Gentner TQ. Latent space visualization, characterization, and generation of diverse vocal communication signals. bioRxiv. 2019;doi:10.1101/870311.

30. van der Maaten L, Hinton G. Visualizing High-Dimensional Data using t-SNE. Journal of Machine Learning Research. 2008;9:2579–2605.

31. Louizos C, Swersky K, Li Y, Welling M, Zemel RS. The Variational Fair Autoencoder. In: Bengio Y, LeCun Y, editors. Proceedings of the 4th International Conference on Learning Representations (ICLR); 2016.

32. Ganin Y, Ustinova E, Ajakan H, Germain P, Larochelle H, Laviolette F, et al. Domain-Adversarial Training of Neural Networks. Journal of Machine Learning Research. 2016;17(59):1–35.

33. Moulines E, Charpentier F. Pitch-synchronous waveform processing techniques for text-to-speech synthesis using diphones. Speech Communication. 1990;9(5):453–467. doi:10.1016/0167-6393(90)90021-Z.

34. Vincent P, Larochelle H, Bengio Y, Manzagol PA. Extracting and Composing Robust Features with Denoising Autoencoders. In: Proceedings of the 25th International Conference on Machine Learning (ICML). ICML '08. New York, NY, USA: Association for Computing Machinery; 2008. p. 1096–1103.
