# Supplementary material for: Measuring context dependency in birdsong using artificial neural networks
Source: PLoS Comput Biol. 2021 Dec 28;17(12):e1009707. doi: 10.1371/journal.pcbi.1009707 (PMC8746767; doi:10.1371/journal.pcbi.1009707)
Supplement: S2 Text — (PDF) [file pcbi.1009707.s002.pdf]

Supporting Information

S2 Details on the Transformer language model

Our analysis of context dependency was based on the Transformer language model of a Bengalese finch song and English sentences [1, 2, 3]. This section discusses the model parameters and training procedure we used. The Transformer consisted of six layers with eight attention heads per layer. The dimensionality of the hidden states, including the middle layer of the MLPs, was 512. We adopted the relative position encoding proposed in [3]. The input embeddings of the Bengalese finch syllables were additively combined with the embeddings of the speaker identity. Dropout was applied at the rate of 0.1 to the input embeddings (+ the speaker embeddings for the Bengalese finch data), the output of each Transformer sublayer before the residual connection, and to the attention weights. We trained the Transformer for $20,000$ iterations using the Adam optimizer with a learning rate of 0.001, $\beta_1 = 0.9$, $\beta_2 = 0.999$, and weight decay of 0.01 [4]. The learning rate was updated according to the schedule used in [1] and [2], with $1,000$ warmup iterations. The batch size was 128.

References

[1] Vaswani A, Shazeer N, Parmar N, Uszkoreit J, Jones L, Gomez AN, et al. Attention is All you Need. In: Guyon I, Luxburg UV, Bengio S, Wallach H, Fergus R, Vishwanathan S, et al., editors. Advances in Neural Information Processing Systems 30. Curran Associates, Inc.; 2017. p. 5998–6008.

[2] Devlin J, Chang MW, Lee K, Toutanova K. BERT: Pre-training of Deep Bidirectional Transformers for Language Understanding; 2018. arXiv:1810.04805.

[3] Dai Z, Yang Z, Yang Y, Carbonell J, Le Q, Salakhutdinov R. Transformer-XL: Attentive Language Models beyond a Fixed-Length Context. In: Proceedings of the 57th Annual Meeting of the Association for Computational Linguistics. Florence, Italy: Association for Computational Linguistics; 2019. p. 2978–2988.

[4] Kingma DP, Ba J. Adam: A Method for Stochastic Optimization. In: 3rd International Conference on Learning Representations, ICLR 2015, San Diego, CA, USA, May 7-9, 2015, Conference Track Proceedings; 2015.
