# Supplementary material for: Measuring context dependency in birdsong using artificial neural networks
Source: PLoS Comput Biol. 2021 Dec 28;17(12):e1009707. doi: 10.1371/journal.pcbi.1009707 (PMC8746767; doi:10.1371/journal.pcbi.1009707)
Supplement: S3 Text — (PDF) [file pcbi.1009707.s003.pdf]

Supporting Information

S3 Analysis of context dependency in zebra finch song

This section reports the Transformer-based analysis of context dependency in zebra finch songs. Readers should remember that the unsupervised, speaker-invariant classification of zebra finches' syllables was not as reliable as Bengalese finches' and that the context dependency reported here is dependent on those classification results.

We used the same song data as those used in the unsupervised syllable clustering by the ABCD-VAE. The data consisted of 11,822 sequences of zebra finch syllables (each containing 1–219 syllables, 20.10 syllables on average) and 11,722 of them were used for training the Transformer language model (Table A). The remaining 100 sequences were used to score the predictive performance of the trained model, from which the dependency (SECL) was calculated.

Our analysis estimated the SECL of zebra finch songs as four (Fig A). Just as Bengalese finch songs did, zebra finch songs showed a trade-off between the number of syllable categories and context dependency, except that the seventeen-way classification—which was automatically detected by the ABCD-VAE—showed a greater difference than the ten-way classification. The difference between the model predictions based on the truncated and full contexts became smaller as the number of syllable categories increased (Fig.; $p < 0.001$ according to the linear regression of the loss difference on the number of syllable categories and the length of truncated contexts, both in the log scale).

Table A: The size of the training and test data used in the neural language modeling of zebra finch songs. The "SECL" portion of the test syllables was used to estimate the SECL. The numbers of syllables in parentheses report the incomplete syllables that were broken off at the start/end of recordings, which were labeled with a distinct symbol.

Usage	# of sequences	# of syllables	
		Total	SECL
Training	11,722	234,674	—
(incomplete)		(5,763)	
Test	100	2,936	1,536
(incomplete)		(55)	(49)

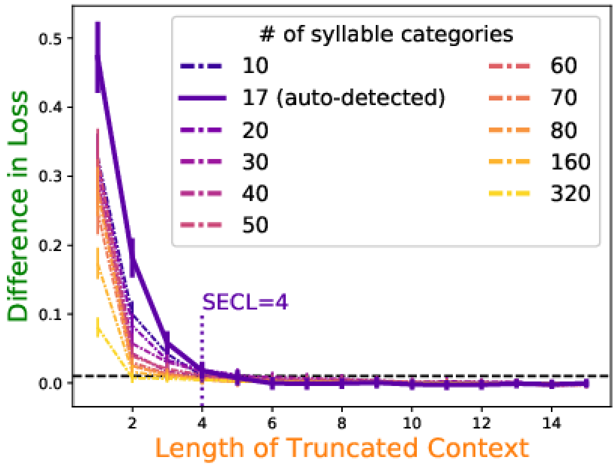

Fig A: The differences in the mean loss (negative log probability) between the truncated- and full-context predictions of zebra finch songs. The x-axis corresponds to the length of the truncated context. The error bars show the 90% confidence intervals estimated from 10,000 bootstrapped samples. The loss difference is statistically significant if the lower side of the intervals are above the threshold indicated by the horizontal dashed line.
