# Supplementary material for: Measuring context dependency in birdsong using artificial neural networks
Source: PLoS Comput Biol. 2021 Dec 28;17(12):e1009707. doi: 10.1371/journal.pcbi.1009707 (PMC8746767; doi:10.1371/journal.pcbi.1009707)
Supplement: S4 Text — (PDF) [file pcbi.1009707.s004.pdf]

Supporting Information

S4 Detailed comparison with the mutual information analysis

This section is a discussion of concrete examples of sequential data where the mutual information metric diverges from the intuitive concept of "context dependency," defined by or related to the memory burden on animal agents that produce/recognize the sequential data. We first introduce a naive definition of mutual information and show that individual-specific tokens can make it constant regardless of their distance in time series. We then discuss a modified version of mutual information that was adopted in previous studies of empirical data, providing some simulation results that demonstrate differences between the mutual information and model-based analyses regarding the detected context dependency (S4.B). It should be noted that recent studies on human language and birdsong did not use mutual information to assess the agent-based context dependency. Instead, they analyzed the decay in the mutual information to diagnose the generative model behind the data [1, 2].

S4.A Problem with individuality

The mutual information I measures the expected divergence between the joint distribution of two tokens, X and X_{+d}, at certain distance d and the product of their marginal probability.

$$\begin{aligned} I(X, X_{+d}) :=& \mathrm{E}\left[\log_2 \frac{\mathbb{P}(X, X_{+d})}{\mathbb{P}(X)\mathbb{P}(X_{+d})}\right] \\ =& \sum_{x}\sum_{x_{+d}} \mathbb{P}(X = x, X_{+d} = x_{+d}) \log_2 \frac{\mathbb{P}(X = x, X_{+d} = x_{+d})}{\mathbb{P}(X = x)\mathbb{P}(X_{+d} = x_{+d})} \end{aligned} \tag{A}$$

Mutual information is zero if X and X_{+d} are independent. It should be noted that this is a pairwise metric, and the other tokens appearing between the two are ignored. For example, X_{+d} can be *conditionally* independent of X given other tokens between them, providing the same information as X_{+d} for the prediction of X, but such relations are not detected by the metric (see Fig Ai).[1]

The mutual information, when naively defined as Eq. A, diverges from the natural concept of context dependency when some tokens encode individual information. Suppose that sequences of tokens are generated by iterating the following procedure:

1. One of two individuals generate a sequence at random (uniformly sample the individual-specific token $s \in \{\mathtt{s}_1, \mathtt{s}_2\}$).
2. Uniformly randomly choose whether a shared or individual-specific token is sampled.
3. Sample one of two shared tokens ($x \in \{\mathtt{a}, \mathtt{b}\}$) or emit the individual-specific token ($= s$).

Then, the probability of each pair of predecessor and follower tokens is as shown in Table A regardless of their distance; we will encounter every possible pair at random except that only one of the two individual-specific tokens is included in a single sequence, and thus, we will never see the heterogeneous pairs, $(\mathtt{s}_1, \mathtt{s}_2)$ and $(\mathtt{s}_2, \mathtt{s}_1)$. Accordingly, the mutual information, if measured globally across sequences, is constant at 0.25. This contradicts the agent-oriented concept of context dependency where the individual agents generate the sequences without referring to the past tokens, and those who read and/or hear the sequences can predict which one of $\mathtt{s}_1$ and $\mathtt{s}_2$ will come next based on their latest occurrence, not further past.

S4.B Algebraic complexity of the corrected mutual information analysis

The particular problem with individual-specific tokens discussed above is not difficult to solve because we can simply condition the relevant probabilities (Eq. A) on the individual that generate each sequence. However,

[1] It is not impossible to assess the conditional effect of mutual information in principle. We may replace all the probabilities in Eq. A with the conditional ones. Such an index, however, would be difficult to estimate in practice because of the exponentially possible sequences of conditioning tokens.

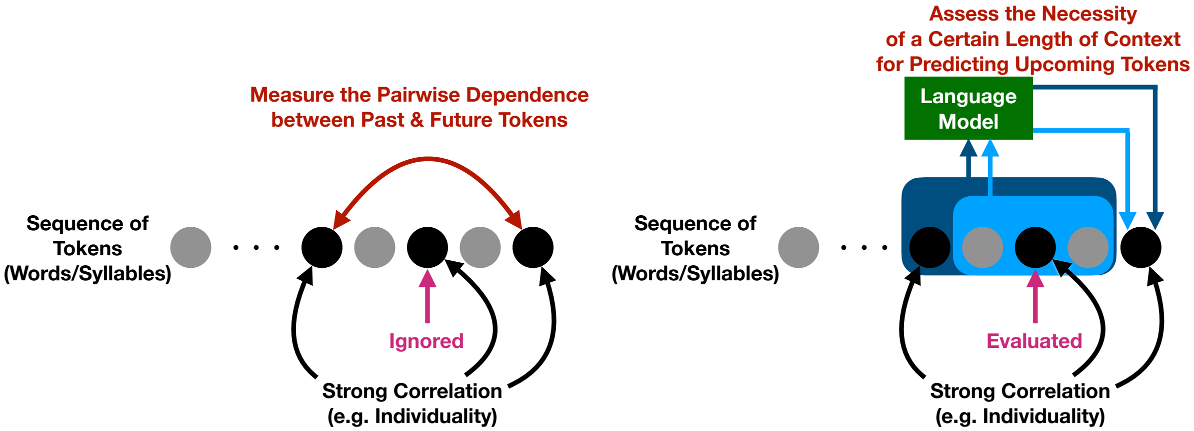

(i) (Naive) Mutual Information Approach

(ii) Language Modeling Approach

Fig A: The analysis of context dependency based on the (i) naive mutual information and (ii) language modeling.

Predecessor (X)	Follower (X_{+d})			
	a	b	$\mathtt{s}_1$	$\mathtt{s}_2$
a	1/16	1/16	1/16	1/16
b	1/16	1/16	1/16	1/16
$\mathtt{s}_1$	1/16	1/16	**1/8**	**0**
$\mathtt{s}_2$	1/16	1/16	**0**	**1/8**

Table A: Probability of each pair of tokens in the same sequence.

the mismatch between the mutual information and the agent-oriented concept of context dependency is not limited to that specific case. In this section, we show that data collected from finite-state automata (FSA), which has been a popular model of Bengalese finch song [3, 4, 5], can yield different mutual information scores despite their identical context dependency from a generative perspective. The simulations in this section are complex and it was not easy to obtain the mutual information from the formal definition in Eq. A. Thus, we used the version of mutual information proposed by Sainburg et al. [2] (termed "SMI" below; cf. the same metric was applied to hierarchical dependencies in human language syntax [6]) for analysis of real birdsong data, which computes an estimated mutual information $\hat{I}$ of data [7, 1] and corrects it with shuffled data $X_{\mathrm{sh}}, X_{\mathrm{sh},+d}$.[2]

$$\begin{aligned}\hat{I}(X, X_{+d}) :=& \hat{S}(X) + \hat{S}(X_{+d}) - \hat{S}(X, X_{+d}) \\ \hat{S} :=& \log_2 N - \frac{1}{N}\sum_{x} N_x \frac{\psi(N_x)}{\log 2} \\ \mathrm{SMI} :=& \hat{I}(X, X_{+d}) - \hat{I}(X_{\mathrm{sh}}, X_{\mathrm{sh},+d})\end{aligned}$$

where N_x counts the occurrences of the category or category pair x, and $N := \sum_x N_x$. Note that $\hat{S}$ estimates the entropy by approximating $\log N_x$ by $\psi(N_x)$, which is considered robust against data sparsity compared to the naive estimation [7]. SMI can capture the concept of context dependency in a better manner owing to the utilization of shuffled baseline in its computation. For example, SMI will be zero for the example with individual-specific tokens discussed in the previous section. The mutual information does not change during the shuffling operation, so the score of the original data is canceled out by that of the shuffled data.

However, there are other cases where SMI diverges from the agent-based concept of context dependency. In the rest of this section, we discuss *periodic* Markov processes [1]. A popular birdsong model (and voice sequences of other animals) is finite-state automata (FSA) [3, 4, 5] (but see [8] and [9] for possible effectiveness of language models beyond the capacity of FSA). FSA transitions among a finite number of states and emits/processes a token associated with the transition. Fig Bi shows a FSA model of a Bengalese finch song that was proposed by Okanoya [4] and is commonly cited in studies about the song syntax [10, 11]. We generated a sequence of $100,000$ tokens from this FSA (with the uniformly random choice between `a` and `c` at the state q_2), and the SMI estimated from this data is shown in Fig C. While the SMI dropped exponentially fast, it is hard to recover the Markovian order of the model, or the context dependency from an agent's perspective: it was not until the inter-token distance was 8 or greater that the SMI went below 0.01 while an agent only needs to remember the last emitted token to correctly simulate the FSA (i.e., the FSA can be simulated by a bigram model; speaking in the language of formal language theory, the generated patterns are 2-strictly locally testable).[3]

Moreover, small modifications to the FSA in Fig Bi can result in completely different SMI scores. Fig Bii removes the branch at q_2 and makes the loop back to q_1 obligatory, while there are still two possible followers of `b` (= `a` and `c`) chosen at random. While this change does not require any extra effort for the data production/processing, and could even simplify the process owing to the reduced number of states, the SMI is now constant around 1.0. Likewise, the small extension shown in Fig Biii, delaying the loop back to q_1 after the choice of `c`, puts the SMI convergence around 1.0. This extension does not change the agent-based context dependency, either as agents can still simulate the extended FSA if they remember the last emitted token and nothing further from the past, preserving the 2-strictly local testability. The non-zero SMI of the non-branching and extended FSAs roots in their periodicity.[4] Taking the non-branching FSA (Fig Bii) for example, we only observe the predecessor-follower pairs, ($\mathtt{a}, \mathtt{c}$), ($\mathtt{c}, \mathtt{a}$), and ($\mathtt{b}, \mathtt{b}$), when they are $2m$ tokens apart ($m \in \mathbb{Z}_+$), and the other patterns occur elsewhere ($2m+1$). Thus, the joint probability of the pairs is different from the product of each member's marginal probability, pulling I (and $\hat{I}$) above zero. On the other hand, this periodicity is broken by the shuffling, creating a difference between $\hat{I}(X, X_{+d})$ and $\hat{I}(X_{\mathrm{sh}}, X_{\mathrm{sh},+d})$

[2] The shuffling operates on each sequence; therefore, tokens in different sequences are not mixed.

[3] Berwick et al. [10] argue that the formal language characterized by the FSA in Fig Bi is not strictly locally testable. This is incorrect because we can test whether each string is a possible outcome of the FSA simply by matching each of its substrings of length 2 to the repertoire. $\{\mathtt{ab}, \mathtt{ba}, \mathtt{bc}, \mathtt{ca}\}$.

[4] Lin & Tegmark [1] prove that mutual information in periodic Markovian processes is characterized by an exponential decay plus a constant bottomline.

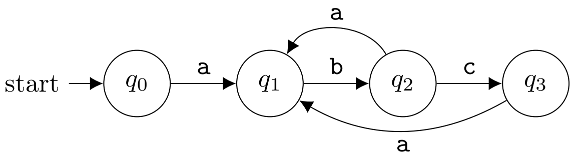

(i) Okanoya [4]

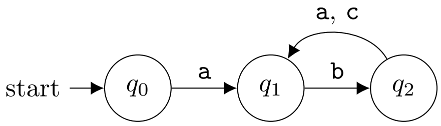

(ii) Non-Branching

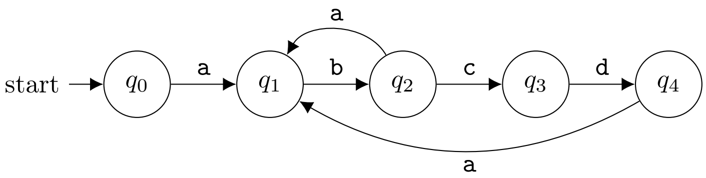

(iii) Extended

Fig B: FSA models of Bengalese finch song. (i) FSA proposed by Okanoya [4]. (ii) A simplified version of (i), removing the transitional branch at q_2 while keeping the two possible emissions, `a` and `c`. (iii) An extended version of (i), delaying the loop back to q_1 after the choice of `c`.

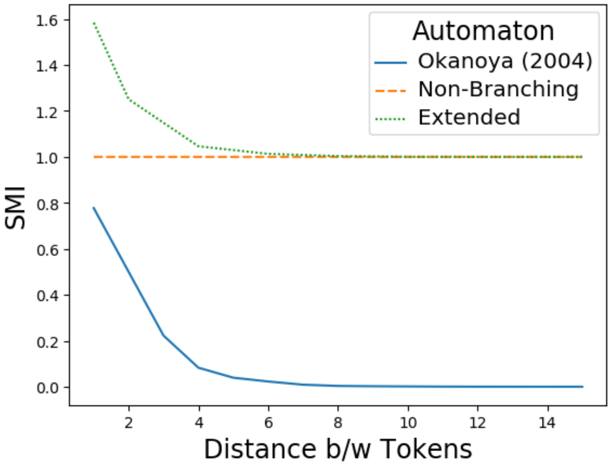

Fig C: SMI of time series data generated by FSA in Fig B.

and keeping the SMI non-zero. Similarly, the extended FSA in Fig Biii goes back to each state two to four steps after it leaves the state. Hence, we never see homogeneous pairs like $(\mathtt{b}, \mathtt{b})$ when their distance is an odd integer.

By contrast, the SECL of the three automata was all 1, which matched the Markovian order of the automata without over-estimating the context dependency. While it is not clear how much periodicity exists in real birdsong and other sequential data in biology, potential differences between the mutual information and model-based analysis should be recognized. Thus, we conclude that mutual information cannot replace the model-based analysis for the assessment of agent-oriented context dependency.

References

[1] Lin HW, Tegmark M. Critical Behavior in Physics and Probabilistic Formal Languages. Entropy. 2017;19(7):299. doi:10.3390/e19070299.

[2] Sainburg T, Theilman B, Thielk M, Gentner TQ. Parallels in the sequential organization of birdsong and human speech. Nature Communications. 2019;10(3636). doi:10.1038/s41467-019-11605-y.

[3] Hosino T, Okanoya K. Lesion of a higher-order song nucleus disrupts phrase level complexity in Bengalese finches. Neuroreport. 2000;11(10):2091–2095.

[4] Okanoya K. Song syntax in Bengalese finches: proximate and ultimate analyses. Advances in the Study of Behavior. 2004;34:297–345.

[5] Kakishita Y, Sasahara K, Nishino T, Takahasi M, Okanoya K. Pattern Extraction Improves Automata-Based Syntax Analysis in Songbirds. Lecture Notes in Artificial Inteligence. 2007;4828:320–332.

[6] Futrell R, Wilcox E, Morita T, Qian P, Ballesteros M, Levy R. Neural language models as psycholinguistic subjects: Representations of syntactic state. In: Proceedings of the 2019 Conference of the North American Chapter of the Association for Computational Linguistics: Human Language Technologies, Volume 1 (Long and Short Papers). Minneapolis, Minnesota: Association for Computational Linguistics; 2019. p. 32–42.

[7] Grassberger P. Entropy Estimates from Insufficient Samplings; 2003. Available from: `https://arxiv.org/abs/physics/0307138`.

[8] Kershenbaum A, Bowles AE, Freeberg TM, Jin DZ, Lameira AR, Bohn K. Animal vocal sequences: not the Markov chains we thought they were. Proceedings of the Royal Society of London B: Biological Sciences. 2014;281(1792). doi:10.1098/rspb.2014.1370.

[9] Morita T, Koda H. Superregular grammars do not provide additional explanatory power but allow for a compact analysis of animal song. Royal Society Open Science. 2019;6(7):190139. doi:10.1098/rsos.190139.

[10] Berwick RC, Okanoya K, Beckers GJL, Bolhuis JJ. Songs to syntax: the linguistics of birdsong. Trends in Cognitive Science. 2011;15(3):113–121.

[11] Miyagawa S, Berwick R, Okanoya K. The Emergence of Hierarchical Structure in Human Language. Frontiers in Psychology. 2013;4:71. doi:10.3389/fpsyg.2013.00071.
