# Supplementary material for: Measuring context dependency in birdsong using artificial neural networks
Source: PLoS Comput Biol. 2021 Dec 28;17(12):e1009707. doi: 10.1371/journal.pcbi.1009707 (PMC8746767; doi:10.1371/journal.pcbi.1009707)
Supplement: S5 Text — (PDF) [file pcbi.1009707.s005.pdf]

Supporting Information

S5 Detailed comparison with the Markovian analysis of context dependency

Previous studies on context dependency in birdsong often modeled song syntax by a Markov process [1, 2]. In this supplementary section, we discuss limitations of this approach, with a specific focus on the method proposed by Markowitz et al. [2].

S5.A Preliminaries

Markowitz et al. [2] estimated the length of context dependency in canary song (whose tokens are chunks of syllables, called *phrases*) based on an algorithm that initially assumes an empty context (i.e, tokens are generated independently of previous outputs) and incrementally checks whether or not longer contexts are needed (under an arbitrary upper bound) [3, 4]. The algorithm can assign different Markov orders to different contexts; for example, production of a binary signal can be dependent on two previous outputs (second order Markov process) when the most recent one is `0` (i.e., $\mathbb{P}(x \mid \texttt{00}) \neq \mathbb{P}(x \mid \texttt{10})$) but not otherwise (i.e., $\mathbb{P}(x \mid \texttt{01}) = \mathbb{P}(x \mid \texttt{11})$). The necessity of a longer context is judged based on two types of probabilities: the joint probability of the context, $\mathbb{P}(x_t \mid x_{t-L}, \dots, x_{t-1})$, and the conditional probability of a next token x_t given the context, $\mathbb{P}(x_t \mid x_{t-L}, \dots, x_{t-1})$. The joint probability must be above a threshold θ_{joint}, filtering out infrequent contexts as negligible. Then, the conditional probability based on the non-infrequent contexts are thresholded ($\mathbb{P}(x_t \mid x_{t-L}, \dots, x_{t-1}) \geq \theta_{\text{trans}}$), and it must also be substantially different from the probability of the same successor token conditioned on the shorter context ($\mathbb{P}(x_t \mid x_{t-L}, \dots, x_{t-1})/\mathbb{P}(x_t \mid x_{t-L-1}, \dots, x_{t-1}) \geq r \vee \mathbb{P}(x_t \mid x_{t-L}, \dots, x_{t-1})/\mathbb{P}(x_t \mid x_{t-L-1}, \dots, x_{t-1}) \leq 1/r$). Contexts passing these tests are considered necessary.

Markowitz et al.'s algorithm has two major difficulties in practical applications. The first is the estimation of the joint and conditional probabilities. The number of possible contexts grows exponentially as they get longer, and fewer observations of each become available. Thus, we cannot naively estimate a distinct probability distribution per long context due to the data shortage and some generalizations over different contexts are needed. In classic Markovian modeling, contexts were often generalized according to their suffix substrings so that the full order conditional probability was estimated from lower order statistics [5, 6, 7, 8]. Markowitz et al.'s algorithm is also one such example wherein unnecessarily long contexts are reduced to their suffixes. Even using such advanced techniques, however, Markovian models were only able to scale up to a few order; for example, even Google's language model—exploiting their big corpora—was based on five-grams (i.e., fourth order Markov) [9]. Similarly, Markowitz et al. estimated the dependency length in canary song as seven, but this was the upper bound set for the algorithm to run. By contrast, recent models based on artificial neural networks represent discrete contexts in a continuous-valued space, wherein generalizations across contexts are made more flexibly [10, 11]. This innovation made it possible to process long context dependencies that potentially range over hundreds of tokens [12, 13].

The second problem with Markowitz et al.'s method is the difficulty in tuning hyperparameters. As introduced above, the algorithm is parameterized by three thresholds ($\theta_{\text{joint}}, \theta_{\text{trans}}, r$) as well as the upper bound L_{MAX} on the possible Markov orders. As we will demonstrate in the next section, different settings of these parameters lead to completely different results and no lesson about their optimization is provided in the literature. Here, our Transformer-based estimation of context dependency has an advantage of robustness; in the next section, we will recover the correct dependency length behind simulated data *using exactly the same hyperparameters as in our birdsong analysis.*

S5.B Comparison using simulated data

This section demonstrates the problems with the Markovian estimation of context dependency and advantages of our proposed method based on Transformer language modeling, using simulated data. The data are generated by a delayed Markov process, which is schematized in Fig A; each token is sampled conditioned on the k-th most recent token in the context (and nothing else); initial k tokens are i.i.d. We adopted the uniform distribution over 37 symbols (= the number of Bengalese finch syllable categories estimated by the ABCD-VAE) for the initial i.i.d. sampling. The transitional probabilities was uniform over three symbols that were randomly selected from the 37 symbols for each condition at the beginning and fixed throughout

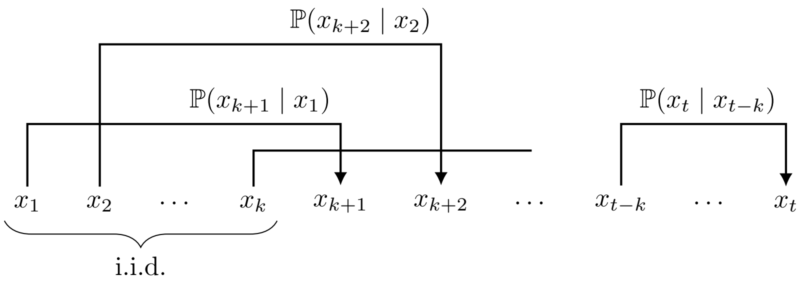

Fig A: Schematic diagram of the Markov process with k-step delays.

Table A: Maximum length of effective contexts detected by the Markovian method. The algorithm was run using two different sets of parameter values, one adopted by Markowitz et al. [2] and the other by Bejerano & Yona [4].

Delay (k)	Maximum Context Length Detected	
	Markowitz et al. [2]	Bejerano & Yona [4]
2	0	5
4	0	3
8	0	3
16	0	3

the sampling process. We set $k = 2, 4, 8, 16$, and for each k, we sampled 10,000 sequences as training data, each of which consisting of 128 tokens, and 100 sequences of the same length as test data (only used in our Transformer-based method).

The maximum Markovian order for Markowitz et al.'s method was set as $L_{\text{MAX}} = 20 > k$. For the other three parameters, we examined two sets of values used in the literature:

- Markowitz et al. [2]
  - $\theta_{\text{joint}} = 0.007$
  - $\theta_{\text{trans}} = (1 + 17.5) \times 0.01 = 0.185$
  - $r = 1.6$
- Bejerano & Yona [4]
  - $\theta_{\text{joint}} = 0.0001$
  - $\theta_{\text{trans}} = (1 + 0) \times 0.001 = 0.001$
  - $r = 1.05$

The maximum length of contexts that were judged as necessary under each of the two settings is reported in Table A. When we adopted Markowitz et al.'s parameters, no effective context was detected. Their threshold for the joint probability, θ_{joint}, was so large that only singleton contexts were qualified. For the same reason, context dependency was underestimated under Bejerano & Yona's settings [4] for $k = 4, 8, 16$; long contexts were not probable enough to pass the threshold. Note that naively decreasing θ_{joint}, and thereby being more tolerant for infrequent conditions, does not lead to the true dependency. This was evidenced by the $k = 2$ example, whose dependency length was overestimated under Bejerano & Yona's settings. Given these results, we conclude that the Markovian estimation of context dependency is not robust.

In contrast to the Markovian estimation, our proposed method based on Transformer language modeling correctly recovered the true dependency length of the delayed Markov process for $\forall k = 2, 4, 8, 16$ (Fig B). The difference between the truncated- and full-context predictions of the test data by the Transformer language model vanished when the length of the truncated context matched k (i.e., the SECL was equal to k). Note that we used the same hyperparameters across different datasets, including birdsong, suggesting that the proposed method is more robust than the previous Markovian estimation.

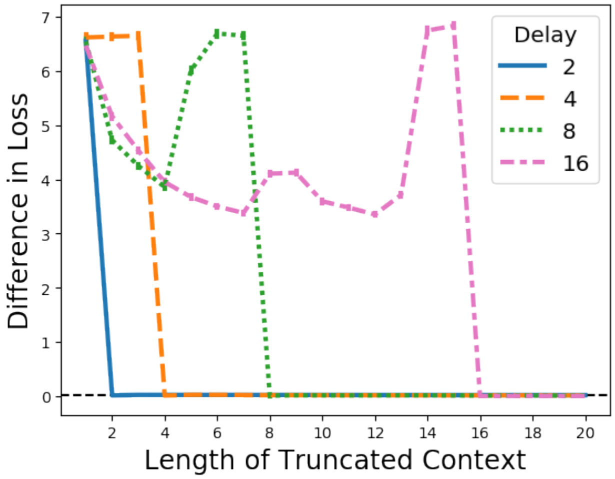

Fig B: The differences in the mean loss (negative log probability) between the truncated- and full-context predictions of time series generated by a k-step delayed Markov process ($k = 1, 4, 8, 16$). The x-axis corresponds to the length of the truncated context. The error bars show the 90% confidence intervals estimated from 10,000 bootstrapped samples. The loss difference is statistically significant if the lower side of the intervals are above the threshold indicated by the horizontal dashed line.

References

[1] Katahira K, Suzuki K, Okanoya K, Okada M. Complex Sequencing Rules of Birdsong Can be Explained by Simple Hidden Markov Processes. PLOS ONE. 2011;6(9):1–9. doi:10.1371/journal.pone.0024516.

[2] Markowitz JE, Ivie E, Kligler L, Gardner TJ. Long-range Order in Canary Song. PLOS Computational Biology. 2013;9(5):1–12. doi:10.1371/journal.pcbi.1003052.

[3] Ron D, Singer Y, Tishby N. The Power of Amnesia: Learning Probabilistic Automata with Variable Memory Length. Machine Learning. 1996;25(2):117–149. doi:10.1023/A:1026490906255.

[4] Bejerano G, Yona G. Variations on probabilistic suffix trees: statistical modeling and prediction of protein families . Bioinformatics. 2001;17(1):23–43. doi:10.1093/bioinformatics/17.1.23.

[5] Katz SM. Estimation of Probabilities from Sparse Data for the Language Model Component of a Speech Recognizer. IEEE Transactions on Acoustics, Speech, and Signal Processing. 1987;35(3):400–401.

[6] Kneser R, Ney H. Improved Backing-off for N-gram Language Modeling. In: Proceedings of the IEEE International Conference on Acoustics, Speech and Signal. vol. 1; 1995. p. 181–184.

[7] Goldwater S, Griffiths TL, Johnson M. Interpolating Between Types and Tokens by Estimating Power-Law Generators. In: Weiss Y, Schölkopf B, Platt JC, editors. Advances in Neural Information Processing Systems 18. Cambridge, MA: MIT Press; 2006. p. 459–466.

[8] Teh YW. A Hierarchical Bayesian Language Model Based on Pitman-Yor Processes. In: Proceedings of the 21st International Conference on Computational Linguistics and the 44th Annual Meeting of the Association for Computational Linguistics. ACL-44. Stroudsburg, PA, USA: Association for Computational Linguistics; 2006. p. 985–992.

[9] Michel JB, Shen YK, Aiden AP, Veres A, Gray MK, null null, et al. Quantitative Analysis of Culture Using Millions of Digitized Books. Science. 2011;331(6014):176–182. doi:10.1126/science.1199644.

[10] Bengio Y, Ducharme R, Vincent P. A Neural Probabilistic Language Model. In: Leen TK, Dietterich TG, Tresp V, editors. Advances in Neural Information Processing Systems 13. MIT Press; 2001. p. 932–938.

[11] Bengio Y, Ducharme R, Vincent P, Janvin C. A Neural Probabilistic Language Model. Journal of Machine Learning Research. 2003;3:1137–1155.

[12] Khandelwal U, He H, Qi P, Jurafsky D. Sharp Nearby, Fuzzy Far Away: How Neural Language Models Use Context. In: Proceedings of the 56th Annual Meeting of the Association for Computational Linguistics (Volume 1: Long Papers). Melbourne, Australia: Association for Computational Linguistics; 2018. p. 284–294.

[13] Dai Z, Yang Z, Yang Y, Carbonell J, Le Q, Salakhutdinov R. Transformer-XL: Attentive Language Models beyond a Fixed-Length Context. In: Proceedings of the 57th Annual Meeting of the Association for Computational Linguistics. Florence, Italy: Association for Computational Linguistics; 2019. p. 2978–2988.
