# Supplementary material for: Measuring context dependency in birdsong using artificial neural networks
Source: PLoS Comput Biol. 2021 Dec 28;17(12):e1009707. doi: 10.1371/journal.pcbi.1009707 (PMC8746767; doi:10.1371/journal.pcbi.1009707)
Supplement: Attachment — Submitted filename: response_letter.pdf [file pcbi.1009707.s006.pdf]

Response Letter

We express our appreciation to the editor and reviewers for their helpful and insightful comments on our manuscript. We addressed all of them in a point-to-point manner in the following letter, and have revised the manuscript in accordance.

In the revised manuscript, we newly assessed how the context dependency interacts with the number of clustered syllable categories. The clustering results were also evaluated quantitatively in more depth using additional baseline and topline clustering methods. We added a new analysis on zebra finches' song data as a comparison to Bengalese finch songs to assess the generality/limitations of our method.

We have withdrawn our claim on the comparison with human language syntax. Such birdsong-language comparison contains a potential problem in token segmentation (i.e. we can not define the best "unit" among birdsong and language) as pointed out by several reviewers, even though our method provides a consistent comparison among discretized sequences after the segmentation. Accordingly, we changed the title of our manuscript as "*Measuring context dependency in birdsong using artificial neural networks*".

Please note that at least two reviewers wrote their comments before reading the supplementary document (as noted by themselves) since we have missed attaching the document at the beginning of the review process, and have sent it to the editorial office later. We attached a revised version of the supplementary information in this submission. We are grateful if the reviewers could have an opportunity to take a look at the document before the final decision on our manuscript.

We believe that the revised manuscript is now suitable for publication in *PLOS Computational Biology*.

Sincerely,
Ryosuke O. Tachibana,
on behalf of the co-authors

Reviewer #1:

> *This paper by Morita and colleagues attempt to quantify long-range statistical dependencies in Bengalese finch songs, comparing this dependency structure to English sentences. They find that, using higher-capacity neural network-based models in place of traditional n-gram and Markov models, they are able to successfully measure dependencies in the eight syllable range, still much less than English sentences but comparable to English syntatical dependencies.*
>
> *This is an interesting contribution to a pair of very long-running scientific questions: 1) to what extent are sequential behaviors like birdsong valid precursors or models for human speech? and 2) how should these complex sequences be modeled mathematically. In my view, the paper makes a somewhat tangential contribution to the former and a solid contribution to the latter.*
>
> *I found the modeling and its interpretation reasonable, though I have a few clarifying questions. Unfortunately, the full packet I downloaded did not contain the supplementary information with modeling details, so while I suspect that the methods are all reasonable, I would like to review that material before I sign off on publication.*

Thank you for your constructive comments. Please find our point-to-point response to your questions/comments below.

In the meantime, we apologize that the supplementary materials were not accessible at the beginning of the review process. We have submitted the supplementary document later on after receiving notification from the editorial office. In this resubmission, we attached a revised version of the supplementary information. We would really appreciate it if you could take a look at it and give us any comments.

> *Apart from this, I have only minor questions and concerns:*
>
> *Minor points:*
> *- It's a bit of a misnomer to say that a Dirichlet prior is "statistically optimal" (l. 91). There are many possible definitions for what constitutes optimality. I might suggest "statistically principled." It is possible to both under-cluster and over-cluster with the Dirichlet prior, depending on what one wants to do, and there's no right answer. Thus, I expected to see the choice of Dirichlet prior defended a little more robustly. I also realize that some of this information was relegated to the supplement, which I will be glad to read.*

Thank you for your suggestion. We agree that optimality is hard to evaluate, especially without the existence of ground truth classifications. Following your recommendation, we rephrased the descriptions of the Dirichlet prior as “statistically principled” or “statistically grounded” throughout the manuscript.

- One thing I'm a bit unclear on: I like the choice of model architecture and the dissociation between bird ID and syllables, but it's unclear to me why the sequence of syllables needs to be discrete tokens and not continuous embedding vectors. Other than alignment with natural human language, why do the syllables need to be indexed by integers and not locations in R^d?

Thank you for your question. The main reason behind syllable classification is to enable our language model to handle non-acoustic, syntactic ambiguity in sequence processing. In human language modeling, for example, “bag” and “wallet” should have more similar syntactic/semantic distributions than “bag” and “beg”, while the latter pair is acoustically similar. Similarly, there is no guarantee that randomness in birdsongs is limited to acoustic ambiguity, so it is not appropriate for a model to make pinpoint or Gaussian predictions about upcoming syllables’ features. By contrast, categorized syllables no longer encode acoustic similarities/differences, and a language model can freely represent syntactic ambiguity among acoustically dissimilar syllables when combined with an output module based on a categorical distribution. This is why we employed the discretized tokens instead of the continuous embedding vectors. Recent machine learning studies on speech/music generation adopt a similar approach as well, and most of the previous studies on birdsong also ran their Markovian or other more traditional models on discrete categories. These suggest that our approach is standard in the field. We clarified these advantages of syllable classification regarding language modeling in the revised manuscript. See the section “Unsupervised, individual-invariant classification of song syllables” (under the Result).

In the meantime, the revised manuscript discusses context dependency of Bengalese finch song based on more fine/coarse-grained classifications than the auto-detected clustering by the ABCD-VAE, and we found that the fine-grained classifications of syllables shorten the context dependency. Fine-grained classifications encode minor acoustic variations among syllables and their successful predictions place greater importance on more local information. We consider that these results provide a deeper understanding of the types of information available from the context (i.e., major patterns encoded in longer context than minor variations) than directly processing the entire acoustic features.

- Another robustness question: How strong is the clustering? Might long-term dependencies be indicative of correlated variability within the bout? Could a faster or slower bout, a softer or louder bout, lead to long-term dependency if the data are over-clustered? Some of these nuisance variables may represent long-range correlations even when bird ID is controlled for. If I were to ask for any additional controls to be run, it would be these.

The robustness of the clustering against variations in amplitude/duration was dependent on syllable categories. We assessed the relation between amplitude/duration of syllables and their probability of maximum-a-posteriori (MAP) syllable categories (see section S1.6 of the supplementary information). The result showed no consistent pattern that the classification probability decreases (i.e., the model gets less confident) as the amplitude/duration diverges from the mean; some syllable categories tended to assign a greater probability to greater or

smaller amplitude/duration while the others were more robust against amplitude/duration differences.

Overall, the Pearson correlation coefficient between the MAP classification probability and the deviation of the amplitude/duration from the median (all in log scale) was small: -0.0791 (vs. amplitude) and 0.0559 (vs. duration) for Bengalese finch; 0.1217 (vs. amplitude) and 0.0328 (vs. duration) for zebra finch. It is also certain that none of the detected syllable categories was purely characterized by amplitude nor duration; syllables having the same amplitude/duration were assigned various classification probabilities, indicating the existence of other acoustic factors that affect the classification.

Additionally, we have tried to artificially manipulate the amplitude and duration of the syllables and test whether such modification changes the classification from the original. Unfortunately, the manipulation of duration (by PSOLA algorithm) did not produce naturalistic syllables (e.g., some spectral bands in spectrograms were blurred). And hence, this approach was not informative to assess the robustness of the proposed method against variations in duration: the classification results were either the same as or different from the original *regardless of the duration of the modified syllables* (e.g., even those having the same duration as the original were assigned a different category). Thus, we did not report this assessment in the revised manuscript.

The supplementary section also acknowledges that researchers may exploit denoising autoencoders to ignore some specific aspects of syllable acoustics for robust classification.

> *Even more minor points:*
> *- Figure 2b: It's hard to tell much from this figure. Might the rows and columns be ordered in some way (most to least used for categories?) in a manner that makes it more apparent which categories are most typical and which individuals show most variety?*

Following your suggestion, We sorted the syllable categories and individual birds by frequency. We also plotted the number of birds/categories covered by each category/bird.

> *- Figure 2c: It's pretty hard to make sense of some of these spectrograms as syllables (e.g., 2, 3). Could the authors perhaps plot the nearest real-data neighbor of these median generated spectrograms?*

In the revised manuscript, we replaced the median spectrograms with the MAP syllable of each category (now renumbered as Fig. 2B). They are the closest to the reference points of the categories represented in the trained model and, thus, show *typical* spectral patterns of each category.

> *- Figure 2e: This is a nice comparison, and it seems clear that giving individual IDs to the seq2seq model does a decent job of correcting for this information. The authors might also consider citing some of the work on similar ideas from the literature, including, e.g., the variational fair autoencoder or domain adaptation, which often want to model some commonality across individuals.*

Thank you for reminding us of the other individual-normalization methods. The revised manuscript discusses them in the Discussion section. In principle, those other normalization techniques can be combined with our discretization-based method.

> *- There is a typo in the last line of the caption to Table 1.*

Thanks, this typo was due to inappropriate LaTeX typesetting. We corrected the typesetting and also made a major revision in Table 1. We double-checked that similar problems are not included in the revised manuscript, though.

> *- The authors note (ll. 247-48) that their estimates of dependency length are unlikely to be model-limited. It is hard to assess this is a rigorous way, but their results, even if they are only a lower bound, are still valuable as such.*

We rephrased the description as "less likely to be upper-bounded by the model limitations and provides a more precise estimation (or at least a tighter lower-bound) of the real dependency length in a birdsong than previous studies", emphasizing our relative achievements compared to Markovian studies.

> *- Why is the train/test split so different between the English and finch data sets in Table 2?*

First of all, the revised manuscript removed the comparison between birdsong and English sentences, following suggestions from Reviewer #2 and #3 that the comparison is not well-grounded unless correspondence between the two data is supported from various perspectives (e.g., correspondence between syllables and words as token units), and thus, there is no longer unevenness between the two types of data regarding the training-test split. Nevertheless, we still held out only 100 birdsong sequences for the test portion in the revised manuscript. This is because birdsongs include more tokens (or syllables) than English sentences, and also because we can perform the analysis of context dependency based on 4,918 syllables. Accordingly, we decided to use most of the available data for training to make the model as reliable as possible.

> *- Details on the bootstrap method for ll. 482-484? This is also in the supplement?*

We clarified the bootstrap method in the main text of the revised manuscript. The bootstrapping was performed *after* the predictive probability of test tokens was computed, so there was no perturbation in the available contexts or any other factors affecting the language model. That is, we just estimated the distribution of the mean difference between model predictions based on the truncated and full contexts by resampling the pre-computed prediction differences of the test tokens.

Reviewer #2:

> *This paper seeks to compare context dependency in the songs of Bengalese finches and human speech. The authors provide a method for unsupervised clustering of BF songs, then use a transformer network approach to predict song element categories based on context from sequences of different lengths. Deviations from full-length context are used to define the “statistically effective context length” (SECL). The authors report that BF song has and SECL of about 8 elements, beyond which context no longer exerts an effect. English lemma sentences have an SECL greater than 10, whereas English sentences relabeled with parts-of-speech tags have an SECL of 5.*
>
> *Overall, I think the paper can be an interesting contribution to the growing literature on long-range dependencies in birdsong, but there are a number of concerns that need to be addressed in any revision. First, the methods need to be more explicitly documented. Both the ABCD-VAE and Transformer network approach are potentially attractive methods for understanding birdsong. However, we don’t learn very much about it either in terms of their performance and robustness in the context of different datasets, or their performance relative to other published methods with similar goals. Second, the ties to human language need to be tempered significantly, if not dropped entirely from the paper. There are a range of conceptual problems in the comparison between songs and speech as presented, and the authors overstate several of the comparisons. Finally, it is not entirely clear how to interpret the present results in the context of past work on the explicit modelling of songs using markov-based and information theoretic approaches. Ideally, contrasts with the published work would done be in quantitatively rigorous ways, befitting the PLOS Comp bio venue.*

Thank you for all your insightful comments and suggestions. Please find our point-to-point response to your questions/comments below.

> *Major concerns:*
> *The ABCD-VAE is very nice and potentially a useful novel method, but its utility as a general method relative to other available techniques is not explored. In particular, recent work shows that BF is not a particularly challenging species to cluster using spectro-temporal acoustics compared to other songbirds and especially to mammals (paper ref 37). Understanding how this method fares in other species, or in more challenging cases in Bengalese finches (if they exist), would be useful. Likewise, a comparison to at least one other unsupervised method would be useful.*

Thank you for your suggestion. First of all, our major contribution regarding the classification of Bengalese finch syllables was the removal of individual variations, which have not been addressed in depth in previous studies (including those on Bengalese finches). Even though

syllable classification is relatively non-controversial compared to other species *when data from each individual bird are analyzed separately *, alignment of syllable categories across individuals is non-trivial in naive acoustic feature spaces. In the revised manuscript, we clarified our contribution (see the Result section) with emphasizing the speaker-normalization effect of the proposed method and also its importance in the main analysis of context dependency; combining data across individuals is crucial to obtain a big dataset which is necessary for training the deep learning-based model.

And following your suggestion, we compared our proposed method with a non-end-to-end clustering method, based on the canonical, continuous-valued VAE and the Gaussian mixture model. This clarified the speaker-normalization effect of the proposed method; naive clustering based on continuous-valued features detected much more syllable categories (128+) that are specific to each individual bird.

In the meantime, the revised manuscript studies zebra finch data in addition and discusses its differences with Bengalese finch results. It turned out that speaker-normalized classification of zebra finch syllables was not successful (or at least less interpretable) compared to the Bengalese finch result. While the proposed method did find common syllable categories shared across different individuals, visualization of category-mate syllables did not provide an easy, intuitive interpretation like “major spectral patterns”. Specifically, complex syllable structures of zebra finches, exhibiting multiple modes of spectral patterns, seem quite unique to each individual even when they were judged as “category-mates” by the model.

> *The critique of Sainburg 2019 (ref 37) in the discussion is not accurate. While it’s true that that study used the MI between pairs of song elements to estimate long range dependencies, the explicit form of the MI decay as a function of dependency distance gives insight to the role of the intervening elements. Specifically, if the decay falls off exponentially, consistent with a Markov model, long distance dependencies require intervening elements (i.e., “everything in the middle”) by definition. It is true that at distances where a power law is exclusively governing decay, the intervening elements can (in principle) be skipped, but the fit of either model and/or their combination is determined by the data, not by an a priori assumption about the role of intervening elements. More importantly, Sainburg et al. 2019 estimate that dependencies shift from being primarily Markovian to primarily power-law distributed at distances of about 24 elements for BFs, which is consistent with the idea that contextual effects as considered in the current paper extend throughout the bout, i.e., over distances considerably longer than the SECL of 8 reported here. The source for this difference is never considered. The authors spend considerable effort in trying to differentiate their results from Sainburg’s on conceptual grounds, but a more convincing approach would be to provide the technical detail necessary to understand how their approach is, as they claim, measuring different components of sequential dependencies in BF songs (as opposed to measuring the same components with different sensitivity). The authors reference a supplementary methods and discussion section, but I could not find either. In particular, more detail for the Transformer model is required. I am not an*

expert in these attention-based approaches, but my understanding is that their primary advantage over recurrent networks in seq2seq tasks is their parallel-izability for training. Since RNN's can model hierarchical structure in sequences (and thus "skip" -or not- intervening elements to find dependencies) whether or not the Transformer network used here behaves similarly needs to be made clear. To do this, it may be necessary to create synthetic datasets that vary in known ways along the dimensions that they argue are relevant to their results. It would also be very helpful to know more about how changing network parameters effects the results.

First of all, we apologize that the supplementary information, wherein we provided concrete examples that demonstrate differences between the model-based and MI-based analyses, was not successfully submitted. We attached the revised version of the supplementary information and we would really appreciate it if you could take a look at it and give us further comments.

The revised manuscript acknowledges the longer context dependency detected by Sainberg et al. and discusses possible factors responsible for the difference. First, long-distance mutual information may not be useful for the specific task of predicting upcoming syllables that defines the model-based context dependency. Specifically, we pointed out that the decay in mutual information can continue beyond the model-based context dependency (providing concrete examples in the supplementary information). We also noted that the main analysis of Sainberg et al. used longer sequences as data (concatenating syllables recorded in a single day). They also reported that the bimodality of mutual information decay became less clear in their bout-level analysis of Bengalese finch song.

Also, the revised manuscript no longer focuses on differentiation between our approach and Sainberg et al.'s, which was just misleading and inappropriate without concrete examples in the supplementary information.

Regarding the Transformer language model, one of its advantages is indeed its ability to capture longer context dependency in data than RNNs in practice. RNNs memorize previous tokens by updating their internal state. Accordingly, retrieval of information in the long past takes a long path in the neural network, and it is known that such long network paths make gradient-based learning harder (even though RNNs can represent infinitely long context information in theory). By contrast, Transformer can directly refer to any token in the input at any time step (except to the future under the language modeling task) and learning long-distance dependency becomes easier. There is also accumulating evidence that Transformer represents hierarchical structures of human language in a more interpretable way than RNNs. The revised manuscript introduces these motivations for the use of Transformer in the introductory paragraph of the Result section. Detailed information about free parameters and the training procedure are provided in the supporting information S2.

The analogy to human language/speech is quite weak. To begin with, the two signals are parsed in much different ways: BF song on the basis of acoustic similarity, and speech on the basis of word boundaries. It's not clear what the analog of a word is in BF song, or if it even exists. The

speech exemplars are then further organized and relabeled by heavily supervised methods (either for the lemma sequences or PoS). Thus categories (and category elements) in the two datasets are very different things and any relationships between them are difficult to interpret. To this point, its not clear that the bootstrapping methods applied to estimate ECL from the small BF dataset were effective. Specific claims in the abstract are overly strong and not supported by the data: (1) "…birdsongs have a long context dependency comparable to grammatical structure in human language", (2) "… birdsong is more homologous to human language syntax…". With reference to the first, the SECL as they define it here for human language is one of many emergent properties of grammatical structure. It is a mistake to conflate grammatical structure with a property of that structure. With reference to the second, I think they mean to imply analogy not homology. In any case, the same confusion of logical type exists.

We agree that our comparison between birdsongs and English sentences was not rigorous, and the revised manuscript removed it entirely. Instead, it now reports how more fine/coarse-grained syllable classifications of birdsong syllables relate to the estimated context dependency. We would appreciate it if you could also give us comments on this new analysis.

Relatedly, it is not clear why the authors introduce the idea of "memory durability" in the discussion (p 18), as nothing in the BF results justifies the notion that either the singers or recipients of song are actively aware or perceptually sensitive to the dependencies reported here. I would not be surprised if birds were sensitive to these dependencies, but it's also possible they are not. For example, dependencies might reflect some other, non-cognitive, aspect of the system such as lower-level constraints on motor production. Appealing to the memory components of language involved in syntax and semantics implies an equivalency that is not supported by the results.

We totally agree with your comment that computational models may confuse actual memory burden on real birds and statistical correlations due to non-cognitive activities. The revised manuscript partially addressed this question by studying the relation between the number of syllable categories and detected context dependency. We found fine-grained classification, which encodes minor acoustic differences among syllables, led to shorter context dependency. This indicates that predictions of minor acoustic variations are reflected in the local domain, rather than in distance.

On the other hand, Reviewer #3 let us know that the context dependency on eight syllables detected in our study was quite similar to the experimental results reported by Bouchard & Brainard (2013), who suggested that HVC neurons encoded information from 7--10 syllables in the context. Thus, we consider that our results are still worth discussion from the perspective of memory.

Reviewer #3:

Summary: The sequencing of elements in communication structures, including language and birdsong, can have long-range dependencies. Here, Morita et al. integrate various machine learning techniques to automatically annotate a large corpus of Bengalese finch song and model the long-range dependencies of syllable sequencing. A similar computational model was also used to quantify the context-dependency of English. Consistent with previous studies, the authors demonstrate that syllable sequencing in Bengalese finch song integrates information across many past syllables to influence subsequent syllable sequencing. The manuscript is well-written overall and the end-to-end, unsupervised clustering approach is a useful addition to the range of techniques used for automatic labelling of birdsong.

Thank you for your constructive comments. Please find our point-to-point response to your questions/comments below.

Major concerns:

1. The authors repeatedly try to link their findings dealing with the context-dependency of syllable sequencing in birdsong to word sequencing in English. For example, in the Abstract they write: "We found that the context dependency in the birdsong was much shorter than that in the sentence, but was comparable to the grammatical structure when semantic factors were removed." It is completely reasonable to compute the number of elements in the past that are required to make accurate predictions about the future for birdsong and language, but it is inappropriate to equate the results from the two because it assumes equivalency between the units of analysis. Consequently, such direct comparisons about context length between birdsong and language should be removed.

That being said, comparisons between the current results on the English language corpus and other analyses of language can be informative and interesting. In its current form, there is not much contextualization of the analyses of language.

Thank you so much for your suggestion. We agree that our comparison between birdsongs and English sentences was not rigorous, and the revised manuscript removed it entirely. Instead, it now reports how more fine/coarse-grained syllable classifications of birdsong syllables relate to the estimated context dependency, following your suggestion #2. We would appreciate it if you could also give us comments on this new analysis.

2. There is insufficient information to evaluate the quality of the automated annotation. As the authors are aware, the inferences from their language model critically depend on the symbols put into the model. While the authors provide some measures of similarity between human and machine classification (e.g., Figure 2 and Table 1), more data is required. For

example, it would be useful to provide estimates of the number of unique syllable types in the songs of each individual Bengalese finch as estimated by humans vs. algorithm. If a human rater is asked to annotate the songs of two birds simultaneously, do they come up with the same number of shared vs. unique syllables between birds as estimated by the algorithm?

The revised manuscript reports the range of the number of manual annotation labels used by the human expert as well as that of model-predicted categories (see the penultimate paragraph of the section “Unsupervised, individual-invariant classification of song syllables” and also Table 1 & 2). The quantitative comparison between human annotations and model predictions was also enriched, mainly by providing baseline and topline results besides our main results.

Ultimately, estimates of the extent of context-dependency depend on the number of classes fed into the model. If one used a broader classification scheme (leading to fewer unique syllable types), estimates of effective context length would decrease. Given the spectrograms in Figure 2C, I suspect that modeling data based on human labelling would lead to fewer syllable classes within the corpus; for example, I suspect that a human would cluster together classes 13 & 20, or even all of classes 1-5. While this does not mean that the classifier is “wrong” (just that machine and human classification can lead to different numbers of classes), this could ultimately affect the number of syllables in the past one might need to analyze to accurately predict the identity of the subsequent syllable.

As we noted in response to your comment #1, the revised manuscript focuses on the analysis of the relation between the number of syllable categories and the length of context dependency. We systematically fine/coarse-grained syllable classifications into 10–80, 160, and 320 categories. On the contrary to your prediction, it turned out that the more syllable categories were assumed, the shorter context dependency was estimated. This is because fine-grained classifications encode minor acoustic variations and their predictions place greater importance on more locally available information.

It would be useful for the authors to provide some raw examples of how measures of similarity between human and machine classification were computed. I suspect that a number of readers might not be familiar with how the V-measure is calculated, so providing a concrete example of how this is computed in their dataset would be useful. (see also Minor comment on Cohen’s kappa)

The revised manuscript includes an explanation about the evaluation policy of each metric as well as some toy examples in the Result section. (The exact computation includes conditional entropy, so we kept the math in the Method section, leaving the Result section intuitively interpretable.)

Please also note that we changed the evaluation metric reported in the main text from V-measure to its submetric called “homogeneity” (V-measure is defined by the harmonic mean of homogeneity and another metric called “completeness”). Intuitively, homogeneity

checks whether the category-mate syllables according to the model predictions were annotated with the same manual label and, thus, quantifies the concept of consistency suggested in your minor comments on Lines 113-114. We also report the V-measure and completeness scores in the supplementary information S1.5.

> *3. Related to point #2, one of the fundamental challenges in bioacoustics analyses that is often ignored is segmentation; classification algorithms are only as good as the elements fed into it. I appreciate the authors acknowledgement of this issue in the Discussion section. However, given their approach and use of segmented syllables, more information about how amplitude thresholds were computed and verified are essential here. References to previous work are insufficient, since understanding the accuracy and reliability of this approach is important to interpreting the data.*

Thank you for pointing out this issue. The revised manuscript provides self-contained descriptions about the segmentation in the Method section.

> *4. I do not see how the current data "provide a new piece of evidence for the hypothesis that human language modules, such as syntax and semantics, evolved from different precursors that are shared with other animals." The analyses presented here do not reveal any separate modules for semantics vs. syntax, and many previous studies have already discussed sequence complexities in vocalizations that lack the semantic content of language. This should be removed.*

Thank you again for your suggestion regarding the analysis of English sentences. As already noted above, we removed the comparison between birdsongs and English sentences.

> *Minor concerns:*
>
> *Beginning from the abstract, the authors repeated make the claim that "birdsong is more homologous to human language syntax than the entirety of human language including semantics." The basis of this statement becomes more evident as one reads the paper, but this is almost impossible to understand in the Abstract. Therefore, the authors need to explain this a little more or rephrase in the Abstract.*

Again, we removed the comparison between birdsongs and English sentences and, accordingly, the abstract is also revised entirely. We made our best efforts to clarify the scope and results of this manuscript in the abstract, but we would appreciate it if you find any other problems in the new abstract and let us know them.

Line 23-25. There should be a more extensive list of papers cited here since many studies have used computational methods to assess the long-range dependency of syllable sequencing in birdsong (e.g., Sainburg et al., 2019 and Jin & Kozhevnikov, 2011 to name a few that are missing from this list).

We extended the list of previous studies there. We also cited Jin & Kozhevnikov (2011) and others to point out problems with Markovian modeling of birdsongs at the end of the same paragraph.

Lines 94-96: The classifier used by the authors detected 39 syllable categories. The authors then write that "(t)he syllable repertoire of each bird covered 26 to 38 categories (34.78 ± 3.19)". If I understand this correctly, this indicates that, on average 35 unique labels would be used to annotate an individual Bengalese finches song. My impression is that this is a substantially higher number than one would normally ascribe to an individual Bengalese finch's song. This gives me pause and further emphasizes the need for additional information and concrete examples about the similarity between human and computer classifications. As indicated above, understanding syllable classification is of utmost importance to understanding the results, so the authors should provide more information about this approach.

The revised manuscript reports the range of the number of manual annotation labels over different birds in Table 1 of the main text (5–14 labels used for Bengalese finches), so that readers can easily recognize that model-predictions are more fine-grained than human annotations. We also clarified the total cardinality and speaker-proportion of each model-predicted category in Figure 2C and 3B.

The revised manuscript also reports various baseline and topline clustering results and demonstrates the advantages of the proposed method. Comparison with those baseline/topline methods also revealed that automatic detection of the number of syllable categories generally resulted in overclassification compared to human annotations.

All of the proposed/baseline/topline methods classified syllables into the same or greater number of categories than human annotations. For this reason, we evaluated the alignment of the clustering results with human annotations without penalizing overclassification (by Cohen's Kappa and homogeneity metrics) in the main text. This evaluation focussed on the consistency of clustering results with human annotations (i.e. clustered syllables were annotated with the same label). On the other hand, we reported more comprehensive results in the supporting information (S1.5) by using V-measure and its submetric called "completeness", which penalizes overclassification, as well. We also clarified the intuitive desideratum behind each of the evaluation metrics as well as their mathematical definition.

Please note that the analysis of context dependency also addressed the effect of over-/underclassification in the revised manuscript, as we commented in response to your suggestion #2.

lines 103-106: "A problem with this metric is that it requires two classifications to use the same set of categories while our model predictions and human annotations had different numbers of categories and, thus, we needed to force-align each of the model-predicted categories to the most common human-annotated label to use the metric." Because Cohen's kappa is a widely used metric that is familiar to many readers, the authors should flesh out this approach more an provide more information on the method of forced alignment. Inclusion of a concrete example would be very useful.

We extended the explanation about the force-alignment by including a toy example that illustrates how it resolved the mismatch in the vocabulary used by the model and human annotator.

Lines 113-114: "Hence, our unsupervised clustering of syllables is as reliable as the manual classification by the expert." The analyses in this paragraph do not speak to the RELIABILITY of labelling (which could be interpreted as the consistency with which an approach labels a particular class of syllables as belonging to that class: e.g., the "a" syllable is always classified as an "a" and never as any other syllable class). Please reword or clarify how these measures speak to the reliability of labelling (as opposed to the similarity of classifications across approaches).

Thank you for pointing out this problem. We reworded "reliable"/"reliability" into "consistent"/"consistency" following your suggestion, and replaced the V-measure metric with homogeneity as it better matches the suggested concept of consistency.

Line 115-119: The performance of a classifier on identifying individual birds depends on how distinct the birds' songs are. Given that the acoustic structure of birdsong is learned, the authors should provide a comprehensive summary of the relationship between birds (e.g., were birds siblings or offspring of another bird in the dataset?)

The revised manuscript provides information about the relationship between birds in the Materials & Methods section. All but two Bengalese finches were obtained from the commercial breeders, and the other two birds (indexed as b10 and b20) were raised in our laboratory. One of these self-bred birds (b20) was a son of a purchased bird (b03), and learned its song from the father. No other birds had any explicit family relationship, so the individual-invariant classification of Bengalese finch syllables is not considered exceptionally easy or non-representative due to relatedness among birds in our dataset. Please also note that even the related birds, b03 and b20, exhibited clear individual differences in the acoustic feature space, as shown in Fig. 2.

Related to the Major Concern #1, the title of the section "Birdsong sequence more context-dependent than English syntax" should be changed because of concerns about equating units of analyses across birdsong and language.

Following your suggestion, we removed the comparison between birdsong and English sentences as already reported above. Accordingly, we also revised the title as "Measuring context dependency in birdsong using artificial neural networks".

Line 137-139. Information about minimum number of syllables per bout should be included here. Generally speaking, I suggest that more information in Methods should be incorporated into this part of the results section, since this would help readers assess the validity of the approach.

The revised manuscript reports the average, minimum, and maximum number of syllables included in the input sequences. Please note that we changed the unit of input sequences from bouts to recordings. The new analysis is more akin to modern human language modeling, which does not segment utterances/texts by sentence and processes longer sequences. The new analysis is more non-parametric than the previous bout-based analysis, wherein the bout boundaries were decided by a conventional threshold of 500+ msec intervals between syllables or the existence of recording boundaries. Also, zebra finch songs became too short when segmented into bouts based on the same threshold (25% of the bouts contained only two syllables). Importantly, however, this revision about the sequence unit did not make differences in the context dependency.

We also moved Table 3 (previously indexed as Table 2) to the Result section, following your suggestion that more information should be moved from the Method to the Result.

Line 147: the summary of English analysis should be in a different paragraph.

We removed the analysis of English sentences from the revised manuscript.

Line 156: the statistically effective context length of 8 syllables is surprisingly similar to estimates of temporal integration by HVC neurons in Bouchard and Brainard (2013). The authors should read and reference this paper to draw parallels. Related to this point, including some discussion of neural mechanisms of temporal integration would be useful in the Discussion section to broaden the scope of the paper and provide some experimental "validation" of temporal integration estimates.

Thank you so much for pointing out this correspondence between our study and Bouchard & Brainard (2013). The revised manuscript discusses their work in the Discussion section.

Line 180-183: The authors discuss how the automated approach is more reliable than human labelling. Can the authors please explain this more? (see point above about assessments of reliability). Relatedly, how is "optimality" defined in this context?

Following your suggestion above, we reworded "reliability" with "consistency" in the section "Quantitative evaluation of syllable classification". On the other hand, the statement at issue in the Discussion section was "more objective and reproducible", and we do not think this entails "reliability". That is, we just mean that the automated classification is free of annotators' perceptual variations and thus is considered more reproducible. Hence, we kept the statement as it used to be, but we are happy to rephrase it if you have some better option.

Regarding "optimality", we received a similar comment on its problem from Reviewer 1 and reworded it into "statistically grounded" or "statistically principled" (based on the Bayesian optimality under the Dirichlet prior).

Line 250: Fujimoto et al. (2011; Neural Coding of Syntactic Structure in Learned Vocalizations in the Songbird") should also be cited somewhere in this manuscript because it provides concrete examples of the history-dependence of sequencing.

Thank you for letting us know about this work. The revised manuscript includes reference to Fujimote et al. (2011) at the discussion about different treatments of heterosyllabic transitions and homosyllabic repetitions.
