# Supplementary material for: Measuring context dependency in birdsong using artificial neural networks
Source: PLoS Comput Biol. 2021 Dec 28;17(12):e1009707. doi: 10.1371/journal.pcbi.1009707 (PMC8746767; doi:10.1371/journal.pcbi.1009707)
Supplement: Attachment — Submitted filename: responseletter.pdf [file pcbi.1009707.s007.pdf]

Response Letter

We express our appreciation to the editor and reviewers for their helpful and insightful comments on our manuscript. We addressed all of them in a point-to-point manner in the following letter, and have revised the manuscript in accordance.

Besides the revisions requested by the reviewers, we also made corrections in the spectrograms in Fig. 2 and 4; the previous version only visualized spectrograms up to the length of the bottom right one and some of them were truncated by mistake. The revised manuscript now reports the entire spectrograms. Please note that this correction was only related to the visualization and none of the quantitative/qualitative results was affected.

We believe that the revised manuscript is now suitable for publication in *PLOS Computational Biology*.

Sincerely,
Ryosuke O. Tachibana,
on behalf of the co-authors

Reviewer #1:

> I appreciate the authors' thoughtful responses to my questions. I think the paper benefits by removing the comparison with English sentences and focusing on the birdsong structure. I was glad to be able to read the supplementary information, which I think is well done and adds much value to the paper.
>
> I have only minor points that I'd ask the authors to consider:
>
> ll. 97-98: Again, this language is problematic.

We removed the phrase at issue, “as a statistical optimum”, from the sentence.

> ll. 110-140: The authors might consider a small schematic figure illustrating this. I think the text description is fine, but a visual illustration would, I suspect, make it easier to grasp.

We added a schematic diagram (Fig. 3) that demonstrates the evaluation of clustering based on Cohen’s kappa coefficient and homogeneity.

> I appreciated the analysis of Section S1.4/Figure S1.3, since I had wondered reading the main text whether the authors had tried to give identity information to the standard VAE as well. (It might be nice to mention that this failed.) Two questions:
> 1) On line 111, when the text says that the "speaker ID" was given to the standard VAE, is this the same embedding vector used in the ABCD-VAE?

The embeddings of speaker IDs were jointly trained with the other modules of the VAE. We clarified it in the revised supporting information.

> 2) Granted, the t-SNE plots do not look clustered at all, but how true is this in the actual latent space? Do clustering metrics also show that clustering performs poorly, or is this just an artifact of the visualization?

We performed GMM clustering on the speaker-normalized Gauss-VAE space, and clarified that the feature space did not suit the clustering purpose, in terms of alignment with human annotations (based on Cohen’s Kappa and homogeneity scores) and speaker perplexity (which was indeed increased when the number of clusters was as moderate as 14 or 37 but stayed below 2 when the number of clusters was auto-detected under the Dirichlet prior, indicating the failure of anonymization). See the supplementary discussion in S1.4 and Table S1.1.

> Table 2: Is the reason for the difference here that zebra finch syllables are just not similar enough to be "speaker de-identified"? That is, are individual birds' repertoires so distinct that it's simply more accurate to use per-bird syllable labels in many cases?

Yes, we consider that zebra finch syllables are more speaker-specific than Bengalese finch syllables, but those individual variations appeared to come from inappropriate segmentation rather than true acoustic variations. Birdsong syllables are usually segmented based on silent intervals between vocalized sounds. Bengalese finch syllables, when identified by this simple segmentation, exhibit uniform spectral patterns over time. By contrast, some of zebra finch syllables include multiple elements of distinctive spectral patterns that are recognized as smaller units, or notes (Cynx 1990, Williams 1990, Williams and Staples 1992, Berwick et al. 2011, Lachlan et al. 2016). In such multi-note syllables (or complex syllables), notes are arranged in a fixed order (without stochastic variations, unlike inter-syllable transitions) but different individuals have different orders. This individual variation in note ordering is not handled appropriately by the seq2seq VAE that assigns a single category or feature vector per syllable without investigating further segmentation of syllable internal structures. Thus, we need a more sophisticated segmentation method for finding note boundaries, just as human speech recognition aims to locate phonemes/(sub)words without relying on silent boundaries.

> SECL results: I remain a bit confused about one aspect here. I appreciate the authors considering these predictions as function of number of syllable categories used, but this clearly complicates the computation of "the" SECL. Indeed, the number of categories used appears to diminish the effectiveness of context, as they report. The authors discuss one possible explanation in ll. 447-455, which is that the larger number of categories simply makes prediction harder, so long-range context matters less. I'm curious about another possibility, though: could it be that using a finer-grained quantization of the existing syllabless, retains more information about previous context (particularly if syllable characteristics like speed or mean pitch are autocorrelated within a sequence), and so I need fewer steps into the past to retain the same amount of predictive information?

Indeed, our intended interpretation of the results is essentially the same as yours: finer-grained classification encoded minor acoustic variations in the syllables, which were predictable from more local contexts. This interpretation is also supported by a previous study which showed that the acoustic profile of classmate syllables can slightly vary depending on the category of neighboring syllables (Wohlgemuth et al., 2010, *J. Neurosci.*). We clarified our intended interpretation in the discussion.

On the other hand, the same previous study by Wohlgemuth et al. also reported that locally conditioned acoustic variations were not characterized by a single acoustic feature; instead, different combinations of multiple features jointly exhibited local variations depending on syllable categories and contexts. Moreover, we cannot distinguish minor acoustic features for characterizing the locally conditioned variations from major features for defining syllable categories. Pitch (fundamental frequency), for example, was one of the features that jointly exhibited locally conditioned variations in Wohlgemuth et al.'s study, but it has also been

used as a major acoustic feature for defining syllable categories (cf., Tachibana et al., 2015, *J. Comp. Physiol. A*; Tian & Brainard, 2017, *Neuron*). Similarly, the acoustic information encoded in our finer-grained classification did not correspond to a single interpretable feature, but rather was a collection of various features embedded in the latent space.

Reviewer #2:

> I appreciate the authors attention to my earlier concerns. The revised manuscript is improved. I continued to struggle, however, with some remaining points.
>
> Absent the link to language, the paper concerns the long-standing question of how to best characterize temporal structure in birdsongs. The paper introduces two novel applications of methods. The first method (called ABCD-VAE) deals with specific questions of how to cluster song element into singer-invariant categories. The second method (using a Transformer model) deals with how to detect long-range dependencies in sequences comprising those categories. The fundamental result is that context dependencies in BF song can be measured quantitatively out to ranges/distances longer than those observed (previously) with other methods. Unfortunately, the use of the singer-invariant categories is still not well-justified or supported by the analyses, and the alleged improvement enabled by the Transformer model is not justified by direct comparison to other methods on the present (or a standardized) dataset. My specific concerns are detailed below.

Thank you for your constructive comments. Please find our point-to-point response below.

> Justification for the use of individually-invariant categories is not well supported. From the standpoint of wanting to approximate the syntactic relationships for words, I can appreciate the desire to find categories for BF song elements that generalize across singers. But this seems like a holdover from the first iteration of the paper with its (misplaced) focus on comparisons to language. BF song elements are not words, and it is less clear that the categories derived through the ABCD-VAE algorithm are biologically justified or functionally valid, even if they are somehow optimized statistically. Repertoire sharing in general is a complicated issue in songbirds and varies tremendously between species. If there are data on repertoire sharing in BF it should be cited. Absent such, it is equally plausible that there is no such thing as an individual-invariant category for BF song elements, or that there exists a mixture of shared and unique elements (as has been reported in other species) and that sharing varies on an individual bird basis. As the authors now report, larger vocabularies lead to lower SECL. This is not surprising given that total entropy is limited by the finite number of transitions in the training set, but it nonetheless highlights the fact that forcing categorization in any arbitrary way could alter the results. The sample spectrograms show significant acoustic variation within some of the ABCD-VAE derived classes and the actual repertoire size within birds is not clear. Since the set of individually-invariant categories (i.e the intersection of all individual categories) is a subset of any individual's categories, it seems likely that

> Transformer models on bird-specific categories that don’t overfit trivial acoustic differences would give similar (or perhaps) even higher SECL. (SECL for individual’s shouldn’t be lower, since the invariant categories have to exist in an individual’s repertoire and sequential structure is ultimately implemented by individual singers.). If this is the case, its not clear what the invariant categorization adds to the paper. If a different result is observed (after controlling for trivial vocabulary size effects), then the use of the invariant categories might be justified. I should add that I think the existence of individually-invariant categories is interesting, I’m just not convinced that it matters for these sequence analysis here.

Our primary motivation for using a common vocabulary and a single sequence-processing model across birds is related to the data size issue as noted in the manuscript. If we try to build separate models for each individual bird, training must rely on smaller data (amounting to 8,509-67,316 syllables per bird, or even smaller if we hold out more data for individual-specific testing). If we train a single model but use individual-specific repertoires, the model still has to learn syntactic properties of each syllable category from sparse observations. We therefore took the option to build a single sequence-processing model on a shared vocabulary while handling syntactic variations among individuals by providing the model with the speaker ID as background information. Once we collect larger data, however, building individual-specific models is of course worth investigating in future studies.

We do not argue that our syllable classification with ABCD-VAE is supported by the biological reality of individual-invariant categories. The biological reality on whether birds share a common set of possible syllable types in their brain, and use them for acquiring and producing their own songs remains an open question.Nevertheless, syllable categories of different birds appear not to be randomly distributed in the acoustic space but instead exhibit similar patterns across individuals even irrespective of tutor-tutee relations. Such inter-individual similarities in syllable categories could emerge without explicit employment of a shared syllable repertoire in the central nervous system. For example, anatomical similarities among conspecific birds bound individual variations in possible articulatory movements, which in turn lead to inter-individual similarities in acoustical features of produced song syllables. Similarly, the auditory capacity would not vary much within the species, and discriminable acoustic patterns would shape similar syllable repertories across individuals. These possibilities might offer indirect support for our individual-independent classification.

> Transformer model benefits:
> With respect to the sequence analysis, I do think the Transformer model is likely to be a very useful tool and its efficiency in other contexts has been shown. In the present case, however, its not clear whether the reported benefits relative to prior work reflects the model itself or the specific dataset (or, as noted above, the novel classification scheme). To show that the Transformer modle is an improvement, a direct comparison is necessary. This should involve either the implementation of a contemporary Markov-based analyses (e.g. as in Markowitz et al 2013) on the current dataset, or the application of the current Transformer model to a the dataset from the Markowitz or a similar study.

According to the reviewer's comment, we added a demonstration for the advantage of Transformer over Markowitz et al.'s Markovian method in the revised manuscript. Since the ground-truth context dependency of real birdsong is unknown, we here used an artificial dataset so that we can evaluate the two methods based on a known ground truth. Specifically, we employed a delayed Markov process to generate the dataset in which each token was sampled conditioned on the k-th most recent token in the context (and nothing else; initial k tokens are i.i.d). We varied the ground-truth dependency length (equal to the delay represented by k) as k = 2, 4, 8, 16. As a result, we found that Markowitz et al.'s algorithm either under- or overestimated the dependency length while our proposed method correctly recovered all k's. Please see the supporting information S5.

> Minor point:
> The discussion (line 287-288) still references a comparison to human text.

We deleted the phrase mentioning comparison to human text.

Reviewer #3:

> The authors have done an excellent job addressing my concerns and revising their manuscript. I am glad to see that direct parallels to English were removed and that analyses of a different species have been included in the revised draft. While the results about content-dependency (history-dependence) are qualitatively similar to previous studies in Bengalese finches, the approach used by the authors is novel to birdsong and potentially powerful (variational autoencoders combined with speaker normalization and Transformer models). I hope that these scripts will be made available to the scientific community for use in their studies.

Thank you for your constructive comments. We will publish all the code used in this study, and also our Bengalese finch data, which would help further investigation of the birdsong in future studies.

> Medium concerns:
> The number of categories determined by the unsupervised approach is ~3-5X as large as those determined by humans. I believe a previous paper (Sainburg et al., 2020?) similarly report that humans overlook context-dependent variation in syllable structure and indicate smaller estimates of repertoire size than other computational approaches. However, given that human labelling has been considered the "gold standard" for so long, running the Transformer model on human annotated Bengalese finch song would be useful (I don't recall seeing these data in the manuscript, maybe because of the limited number of human annotated songs). Given that they find an inverse relationship between the number of syllable classes and SECL, it seems like a greater SECL would be found in the human annotated data.

Transformer-based analysis of human annotated data is not investigated in this study mainly for two reasons. The first is the limited availability of original annotations (only less than 3% of the entire dataset was annotated), as you suspected. Thus, we would need some supervised machine learning that generalizes the (limited) human annotations to unlabeled data, which is out of the scope of the current study. Secondly, the human annotations are individual-specific, and the total number of syllable categories (= 167) becomes greater than 37. Thus, we are currently unable to test your hypothesis in the intended way, so we leave the investigation for future studies. Note that we will publish all the code used in this study so that other researchers can analyze their own manually annotated data using our method.

> It’s unfortunate that the author’s current attempts at unsupervised classification of zebra finch songs were not particularly successful. Zebra finches are the most commonly studied songbird so optimizing an approach for this species would be particularly impactful. And spectral complexity is not an uncommon feature of animal communication signals, suggesting limited applicability of the current approach. That being said, testing this approach on canary song syntax would be useful given the relative spectral simplicity of canary song syllables and previous studies of context-dependency in canary song. (NOTE: this is not to say that an analysis of canary song is imperative for the publication of this manuscript, just that it would be a welcome addition.)

Unfortunately, we were not able to obtain an appropriate dataset of canary songs for our methods. It is doubtless that canary song is worth investigating under our framework, so we are willing to study it in a future work, once we obtain data. Again, we will publish all the code used in this study, so other researchers can also work on canary and other birds’ songs using our methods.

> Much of the results from the Transformer model of zebra finch song should be excluded from the main text. If syllable classification is unreliable for zebra finches, modelling the sequence structure of unreliably annotated songs can be misleading. It runs the risk of some readers concluding that 4 syllables are required to make accurate predictions about upcoming syllables in zebra finch song. The authors already word this section of the manuscript carefully, but I think they should prune this down even more to simply indicate that (similar to Bengalese finch song) SECL becomes smaller as the number of categories increases for zebra finch song. Related to this point, Figure 4C can be moved to supplementary information and references to an SECL of 4 should be removed from the Discussion.

Following your suggestion, we moved the analysis of context dependency in zebra finch songs to supplementary information S3. The limitations of the reported results were emphasized at the beginning of the supplementary section. We also refrained from the comparison of SECL between Bengalese finch and zebra finch based on the syllable clustering by ABCD-VAE (eight vs. four), and simply reported the estimated statistics (including the relation between the number of syllable categories and dependency length).

Minor concerns:
Lines 218-224: only 16 of the 20 birds are accounted for in this description. Please revise.

We corrected the counts at issue. There were six “moderate agreement” birds rather than two.

Line 388-389. Can the authors confirm whether the canary study cited here analyzed canary syllables or phrases. The time scales of these two types of song descriptions are very different (7 syllables in the past is much shorter than 7 phrases in the past), so this should be clarified and indicated in the manuscript.

We clarified that the song units were phrases in the cited study.
